# Update on lyssaviruses and rabies: will past progress play as prologue in the near term towards future elimination?

**DOI:** 10.12703/b/9-9

**Published:** 2020-11-16

**Authors:** Rodney E Rohde, Charles E Rupprecht

**Affiliations:** 1 Clinical Laboratory Science, Texas State University, San Marcos, TX, 78666, USA; 2 LYSSA LLC, Cumming, Georgia, 30004, USA

**Keywords:** Disease, Elimination, Encephalitis, Lyssavirus, Rabies, Vaccine, Virus, Zoonosis, Rabies

## Abstract

Rabies is an ancient, much-feared, and neglected infectious disease. Caused by pathogens in the family Rhabdoviridae, genus *Lyssavirus*, and distributed globally, this viral zoonosis results in tens of thousands of human fatalities and millions of exposures annually. All mammals are believed susceptible, but only certain taxa act as reservoirs. Dependence upon direct routing to, replication within, and passage from the central nervous system serves as a basic viral strategy for perpetuation. By a combination of stealth and subversion, lyssaviruses are quintessential neurotropic agents and cause an acute, progressive encephalitis. No treatment exists, so prevention is the key. Although not a disease considered for eradication, something of a modern rebirth has been occurring within the field as of late with regard to detection, prevention, and management as well as applied research. For example, within the past decade, new lyssaviruses have been characterized; sensitive and specific diagnostics have been optimized; pure, potent, safe, and efficacious human biologics have improved human prophylaxis; regional efforts have controlled canine rabies by mass immunization; wildlife rabies has been controlled by oral rabies vaccination over large geographic areas in Europe and North America; and debate has resumed over the controversial topic of therapy. Based upon such progress to date, there are certain expectations for the next 10 years. These include pathogen discovery, to uncover additional lyssaviruses in the Old World; laboratory-based surveillance enhancement by simplified, rapid testing; anti-viral drug appearance, based upon an improved appreciation of viral pathobiology and host response; and improvements to canine rabies elimination regionally throughout Africa, Asia, and the Americas by application of the best technical, organizational, economic, and socio-political practices. Significantly, anticipated Gavi support will enable improved access of human rabies vaccines in lesser developed countries at a national level, with integrated bite management, dose-sparing regimens, and a 1 week vaccination schedule.

## Introduction

As reflected by the recent emergence of the novel coronavirus, SARS-CoV-2, such pathogens continue to pose substantial yet somewhat predictable concerns to human health and welfare at a global level. In contrast to more newly appreciated threats, rabies is one of the oldest described infectious diseases, likely with a more ancient pedigree, pre-dating most historical accounts^1^. Rabies and related lyssaviruses continue to cause major mortality in countless mammalian species, including *Homo sapiens*, domestic animals, and wildlife^2^. Although lyssavirus-related mortalities remain uncommon in humans within developed countries, these viruses, and the burden posed in lesser developed countries (LDCs), have captured the attention of scientific, agricultural, and public health communities because of their extreme fatality rate, the highest for any conventional agents, with an estimated human death every 10–15 minutes^3^. Having the capacity to cause severe disease with serious health and economic implications, without efficient treatment yet available, rabies is considered a major neglected viral zoonosis. As a vaccine-preventable disease, the Food and Agriculture Organization (FAO), the World Organization for Animal Health (OIE), and the World Health Organization (WHO) have focused upon an ambitious plan for the global elimination of human rabies mediated via dogs (GEHRD) or ‘Zero by Thirty’ (ZBT) by 2030 (https://www.who.int/news-room/detail/28-09-2019-united-against-rabies-collaboration-celebrates-one-year-of-progress-towards-zero-human-rabies-deaths-by-2030). This plan was perhaps the singular galvanizing event of the early 21^st^ century, in a sea of other notable examples over the past 10 years, that has reset the underpinnings for challenges and success in the field, as currently appreciated^4–19^ (Table 1). The objective of this brief commentary is to provide an update on the recent progress and extant dilemmas related to rabies and to highlight evidence-based opinions on the evolving scenarios forecast to arise over the next “ZBT decade”, illustrated objectively by data provided through the contemporary, peer-reviewed literature, exemplified primarily during the last few years^1–201^.

**Table 1.** Highly notable events in the applied rabies field related to detection, prevention, and control over the past decade.

Item	Reference
Discovery of new lyssavirus species	4
Suggestion of rabies virus adaptation beyond carnivores and bats to other mammals, such as non-human primates	5
Greater appreciation of wildlife reservoirs in previously considered “rabies-free” areas	6
Recognition of additional antigen detection, serological, and molecular tests for very sensitive and specific lyssavirus diagnosis	7
Recognizable shifts from animal culling to mass dog vaccination as a proven management strategy	8
Planning for the global elimination of human rabies mediated via dogs by 2030	9
Greater focus upon local infiltration of wounds with scarce rabies immunoglobulins	10
Availability of human monoclonal antibodies as an alternative to polyclonal rabies immunoglobulin	11
Recommendations on dose-sparing and shorter 1 week human prophylaxis regimens	12
Expansion of the distribution of vampire bats and rabies virus spread	13,197
Support for pre-exposure vaccination for those in remote settings, such as children in communities with a high exposure rate to canine rabies virus and those at risk of vampire bat depredation	14
*In vitro* alternatives to animal testing in the determination of vaccine potency	15
Demonstration of compounds with repeatable *in vitro* anti-rabies virus activity	16
Renewal of interest for the oral vaccination of free-ranging dogs against rabies	17
Elimination of canine rabies in Mexico	18
Expectations of Gavi support for human rabies vaccination	19

## Viral characteristics

Taxonomically, the etiological agents reside in the order Mononegavirales, family Rhabdovirus, genus *Lyssavirus*. Rabies virus is the type-species and most important member of this mono-phyletic genus. Since the 1950s, at least 17 other recognized and proposed lyssavirus species have been described from Africa, Australia, and Eurasia, all of which can be differentiated antigenically and genetically yet cause a clinically indistinguishable encephalitis and not a so-called ‘rabies-like’ disease (https://talk.ictvonline.org/ictv-reports/ictv_online_report/negative-sense-rna-viruses/mononegavirales/w/rhabdoviridae/795/genus-lyssavirus).

The basic viral structure consists of a characteristic bullet-shape and helical symmetry by transmission electron microscopy (https://apps.who.int/iris/bitstream/handle/10665/310836/9789241515153-eng.pdf?ua=1). Ultrastructurally, lyssaviruses may be distinguished from other rhabdoviruses, but not from one another. Virions have a length of about 180 nm (130–300 nm) and a diameter ranging from 45 to 100 nm (with one rounded and a flattened end). The bacilliform or rod-like particles appear hemi-spherical at both ends when mature. Lyssavirus plane projection imagery reveals surface spicules, a host cell-derived envelope, and a nucleocapsid with helical symmetry. This thin fringe of spicules, about 8 nm thick, does not cover the surface of the plane end of the virus. The genome, approximately 12 kb in size, is composed of a negative-sense, single-stranded, non-segmented RNA and contains five transcription units encoding five viral structural proteins (3′-N-P-M-G-L-5′), separated by short noncoding introns, except for a longer, noncoding intergenic G-L region (https://apps.who.int/iris/bitstream/handle/10665/310837/9789241515306-eng.pdf?ua=1). The viral structural proteins include the nucleoprotein (N, ~400 aa), the phosphoprotein (P, ~300 aa, as a cofactor of the polymerase), the matrix protein (M, ~200 aa), the outer surface glycoprotein (G, ~500 aa), and the RNA-dependent RNA polymerase (L, ~2,000 aa). Besides their classically recognized role in structure, receptor binding, membrane fusion, endosome formation, uncoating, encapsidation, transcription, translation, replication, assembly, and release, the viral proteins are also involved in subtle immune evasion and overt disease progression^20,21^. Improving upon much earlier ultrastructural accounts, recent studies using high-resolution imaging, cryo-electron microscopy, crystal structural and functional analyses, proteomic profiling, and molecular modeling have provided unique insights into a finer understanding of dynamic viral and host protein interactions, neurotropism, the underlying regulation of replication and transcription, intracellular transport, and the future development of more rapid diagnostics, novel biologics, and rational anti-viral drug design^22–32^.

## Pathogenesis

In a rabid animal, virions are shed intermittently in the saliva, usually transmitted via a bite and deposited deeply within a wound, eventually access the peripheral nervous system, travel in retrograde fashion, replicate primarily in the central nervous system, and transit gradually to the salivary glands and other tissues, in a well-known, generalizable productive infectious cycle model^33^. Lyssaviruses display a high degree of neurotropism, with a preference for neurons, but non-neuronal cells may also be targeted^34^. For entry, the viral G protein can recognize not just one but several host cell receptors that are highly conserved among a diversity of avian, marsupial, and placental species, albeit at apparently different relative efficiencies and outcomes. For example, the wide-ranging ability to infect neuronal, muscle, and epithelial cells and fibroblasts suggests a rather ubiquitous expression of entry receptors in different tissues^33^. In contrast to some other rhabdoviruses, lyssaviruses are not lymphotropic. Besides neurons, some lyssaviruses can infect specialized neuro-epithelial cells^35^. Nevertheless, viral dissemination within the infected host is facilitated primarily via attachment to, transport by, and replication within neurons^33^. As a quintessential neurotropic pathogen, rabies virus continues to be used experimentally as an anatomical transneuronal tracer, to better understand connectivity within the nervous system^189^.

Viral replication and assembly occur in compartmentalized, sequestered intracytoplasmic “factories”, historically termed Negri bodies, a histopathological hallmark of infection for over a century, although not always detected readily, which limited their diagnostic utility in the face of improved tests^29^. Despite the virulence of rabies, gross and microscopic injuries may appear rather minor and principle pathogenic mechanisms remain unclear. For example, lyssavirus infection of neurons may result in mitochondrial dysfunction, producing oxidative stress and acute degenerative changes of neuronal processes^36^. In addition, one potential host-mediated response implicates a role for the *SARM1* gene in axonal “self-destruction”, impeding viral spread but also with subsequent pathological impacts of neuronal and dendritic cell loss^37^.

Operationally, exposure is defined as transdermal or mucosal contamination with saliva, brain tissue, or other virus-containing substances^2^. Human cases continue to be documented following exposure via these well-recognized routes^38–40^. Incubation periods range from less than a week to greater than a year (i.e. typically shorter, after bites to the head and neck), with most cases presenting within 4–6 weeks of exposure^41^. The few documented occurrences of exceptionally long incubation periods (i.e. exceeding several years) remain poorly understood regarding mechanism, localization, recognition, etc., or whether much delayed prophylaxis would be effective after the initial infection process^2^. Initial illness is characterized by non-specific onset, including fever, headache, dizziness, vomiting, and myalgia. Later, subjects may experience severe encephalitis, including hydrophobia, aerophobia, and photophobia. Patients succumb from cardio-pulmonary dysfunction and complications related to multiple organ failure^42^. Delayed innate immune responses may contribute to pathology^43^. The very few survivors from infection frequently have long-term neurological sequelae^44,190^. Clinical suspicion is heightened after documentation of rabid animal exposure and the onset of compatible signs, but conflicts in adequate laboratory confirmation may confound interpretations^45^.

## Immune responses

Innate immunity plays a critical first-line role in anti-viral host defense and modulation of infection after lyssavirus exposure. Toll-like receptors (TLRs), cytoplasmic ds-RNA, and triphosphate-RNA sensors (among others) are part of the host pattern recognition system, activated after sensing of viral RNA post-infection^186,187^. Activation of TLR3, TLR7, etc., results in a cascade of events, including the production of interferons (IFNs) and interleukins and the induction of initial adaptive immune responses, including the recruitment of B cells and facilitation of germinal center formation^46^. If, after inoculation into a peripheral lesion, local lyssavirus infection is followed by the production of viral RNA, sensed by TLRs and other pathways, leading to the activation of IFN-stimulated genes, induction of IFN, and the incitement of subsequent anti-viral signaling, then how does a productive infection actually ensue? Suppression of anti-viral defense signals, such as for IFN production, could lead to a promotion of viral spread by disrupting both innate and adaptive immunity. Several mechanisms have been described in which viral structural proteins were found to be involved in the blocking of cytokine signaling pathways^47^. In addition, lyssaviruses may restrict G protein expression and reduce its incorporation into mature virions, subverting the activation of antigen-presenting dendritic cells^48^. Such mechanisms constitute a combined viral immune evasion and suppression strategy, supporting overall a more efficient host invasion. Although somewhat ignored, immunity as defined by the induction of virus-neutralizing antibodies (VNAs) against lyssaviruses can be operative and protective in naïve (i.e. unvaccinated human or other animal) hosts^49^. Administration of attenuated, recombinant, and adjuvanted veterinary biologics or high-potency, multi-dose, prime-boosting applications of inactivated human vaccines promote both innate and humoral immunity via antigen-presenting cells, T cell differentiation, the induction of VNA-secreting plasma cells, and long-lived memory B cells^50^.

## Diagnostic applications

Often overlooked, rabies diagnosis has undergone a seeming renaissance of late^2,7,51–53^. Infection is confirmed by the finding of viral antigens, antibodies, amplicons, nucleic acids, or biomarkers in subjects with signs compatible with an encephalitis. When a case of rabies is suspected, management decisions are made that run the gamut from an individual, exposed patient to a programmatic intervention^54^. Hence, laboratory evaluation is critical. Although most testing occurs postmortem in animals, the diagnosis of rabies in humans prior to death (i.e. antemortem) provides definitive diagnosis for infection control, closure for families, identification of others who may have been exposed to the same source, appropriate patient palliation or rare hope, and the opportunity to attempt experimental therapeutic approaches^55^. Prior traditional methods for antemortem and postmortem rabies diagnosis had multiple limitations. Advances in “best fit” technology and understanding of basic viral pathobiology have led to the improvement or design of many more diagnostic options, beyond the 20^th^ century detection of Negri bodies. Such newer assays, augmented by traditional methods, have begun to revolutionize lyssavirus diagnosis across the global landscape. Unfortunately, while there are antemortem methods for confirmation concomitant with encephalitis, there are still no sensible diagnostic tests available for lyssavirus detection prior to onset of clinical disease. Moreover, assay choice, sample selection, protocol adherence, and post-analytical interpretation issues are not unique to lyssavirus diagnostic challenges, as described in detail in the latest WHO monograph of laboratory methods^7^.

Historically, conventional testing included (1) direct microscopic detection of intracytoplasmic inclusions (i.e. Negri bodies) in infected neurons (no longer recommended for routine primary diagnosis); (2) direct fluorescent microscopy (direct fluorescent antibody [DFA]) during postmortem diagnosis (i.e. widely used in animals and humans as a standard test), with direct staining of viral antigens in touch impressions of brain tissues, including portions of the brainstem (i.e. needed for a definitive diagnosis), the cerebellum, or the hippocampus; (3) virus isolation (i.e. usually reserved in research settings or for confirmatory diagnosis when the DFA test gives a weak or uncertain result) with two primary tests being the mouse inoculation test and the rapid tissue culture infection test in MNA cells; (4) rapid rabies enzyme immune diagnosis, an enzyme-linked immunosorbent assay (ELISA)-based technique which detects the viral N antigen; (5) antibody demonstration in the serum (in the absence of a history of vaccination) or in CSF, offering indirect evidence of infection by demonstration of anti-N antibodies or anti-G VNA (i.e. via virus neutralization), including the mouse neutralization test (no longer recommended), the rapid fluorescent focus inhibition test (RFFIT), and the fluorescent antibody virus neutralization test (FAVN).

Over the past decade, a variety of molecular methods, many based on PCR modalities, are increasingly applied to various sample types for human antemortem diagnosis and as a confirmatory test for other samples^7^. Likewise, immunohistochemical methods traditionally applied for rabies diagnosis of fixed, paraffin-embedded tissues are now being leveraged as tools explore for more rapid applications. These tests, many now automated, include (1) the direct rapid immunohistochemical test (dRIT), which is an approximately 1 hour test based on detecting viral N protein in brain tissue; (2) the indirect rapid immunohistochemistry test (iRIT), offering the detection and differentiation of virus variants via traditional light microscopy; (3) the reverse transcriptase polymerase chain reaction (RT-PCR) assay, the most frequently employed molecular method that seeks to detect rabies virus and related lyssavirus RNA; (4) the qPCR-based assays (with varying chemistry and detection kits), allowing for the “real-time” detection and quantification of genome copies, with the advantage of a closed-tube assay for a significant reduction in cross contamination; (5) the Qiagen QIAsymphony SP/AS, in conjunction with quantitative reverse transcription-PCR (qRTPCR); (6) the rapid immunodiagnostic test (RIDT), detecting antigens from postmortem samples and utilized without the need for more sophisticated laboratory equipment, which is based on a lateral flow strip assay in a one-step test that facilitates low-cost, rapid identification of viral antigens; (7) nucleic acid sequence-based amplification (NASBA), which allows the utilization of three enzymes to produce multiple copies of RNA in isothermal conditions; (8) and several other updated assays (e.g. Platelia Rabies II ELISA, a rapid antibody detection test (RAPINA) based on immunochromatography, latex agglutination tests for rabies virus-specific antibodies, proteomics, metabolomics, etc.). These provide multiple options for laboratorians in both developed countries and LDCs.

One additional method, the LN34 pan-lyssavirus RT-PCR assay, represents an idealized candidate test for postmortem diagnostics, owing to its ability to detect RNA across the diversity of the viral genus, high sensitivity, potential for use with deteriorated tissues, and user-friendly design^56^. Providing data from a multi-site evaluation of the LN34 assay in 14 laboratories using 2,978 samples (1,049 DFA-positive) from Africa, the Americas, Eurasia, and the Middle East, high diagnostic specificity (i.e. 99.7%) and sensitivity (i.e. 99.9%) were shown when compared to the DFA test (i.e. no DFA-positive samples were negative by the LN34). The LN34 assay exhibited low variability in repeatability and reproducibility studies, suggesting a new gold standard for centralized laboratories. Once quality control is optimized, utilization of more directed, improved point-of-care diagnostics should range from enhanced surveillance activities in the field to better assessment of exposed patients in the clinic under more real-time conditions in support of better understanding of the underlying disease epidemiology for timely responses^2,7,57,58^.

## Epizootiological insights

As a representative disease of nature, any presumption that rabies is “rare” is a simple fallacy, dependent upon epidemiolocal context and the public/professional reference frame (http://outbreaknewstoday.com/rabies-signs-and-symptoms-exposure-transmission-and-diagnostics-81094/). As lyssaviruses are RNA viruses, an expectation of reasonably high mutation rates in the face of strong purifying selection is the rule for anticipated fixation, adaptation, emergence, and extinctions in the short term and over longer historical periods. Lyssaviruses exist as “ecological ensembles”, metapopulations of distinct species and variants, residing within multi-reservoir mammalian communities in often rapidly changing environments^59^. Such host population–viral assemblages are perpetuated in ecologically diverse urban, rural, and wilderness ecosystems, from the Tropics to the Arctic^60–64^. Despite this broad host spectrum and wide geographic distribution, from a public health perspective, based upon laboratory-based surveillance and epidemiological analyses, today the overwhelming number of human fatalities are still due to rabid domestic dogs, primarily in LDCs^65,66^. By comparison, transmission to humans by rabid wildlife, in both developed countries and LDCs, is much less common^67–76^ (Table 2).

**Table 2.** Documented examples of recent reports of human rabies cases transmitted by wildlife.

Mammal	Locality	Reference
Insectivorous bat	USA	67
Vampire bats	Latin America	68
Wolf	Russian Federation	69
Fox	China	70
Jackals	Bangladesh	41
Raccoon dogs	South Korea	71
Ferret badgers	China	72
Skunk	Mexico	73
Raccoon (or spillover to cat?)	USA	74
Mongoose	Puerto Rico	75
Non-human primates	India	76

Evolutionarily, bats are recognized as the ultimate reservoir of the lyssaviruses^1,2,77^. Despite more than 17 conspecific members, rabies virus appears to be the only lyssavirus with clear reservoir representation among multiple orders of mammals^1,2,78^. Unique to the region, independent rabies virus lineages may be found among multiple bat species throughout the Americas^64,79–81^. Host shifts from engagement of bat rabies viruses to other mammals are suggested by derived variants in raccoons, skunks, and marmosets in the New World^1,2,5,82,83^. Additionally, multiple mesocarnivore variants (much more distantly descended originally from ancestral bat rabies viruses) are now represented by dogs, other wild canids, mustelids, mongoose, etc. in both the Old and the New Worlds^84^. Although most rabies virus variant transmission patterns are intrinsically intraspecific (e.g. dog-to-dog, bat-to-bat, etc.), interspecific spillover infection may occur to practically any bitten mammal, from a veritable alphabet soup of armadillos to zebras^85^. While some spillovers may be amplified by short transmission chains, the majority of these are ultimately dead-end infections, such as to domestic or wild hoofed stock. Perhaps the most extreme example of the latter is the case of dog-jackal-kudu rabies in southern Africa^86^. Transmission of rabies viruses from domestic animals or wildlife to humans is almost always a single term event (i.e. the person dies without a secondary case), except for the rarity of human-to-human infection from tissue/organ transplants^87^. Such instances are examples of the devastating amplifying consequences when rabies is unsuspected, ignored, or mis-diagnosed.

**Figure 1.**
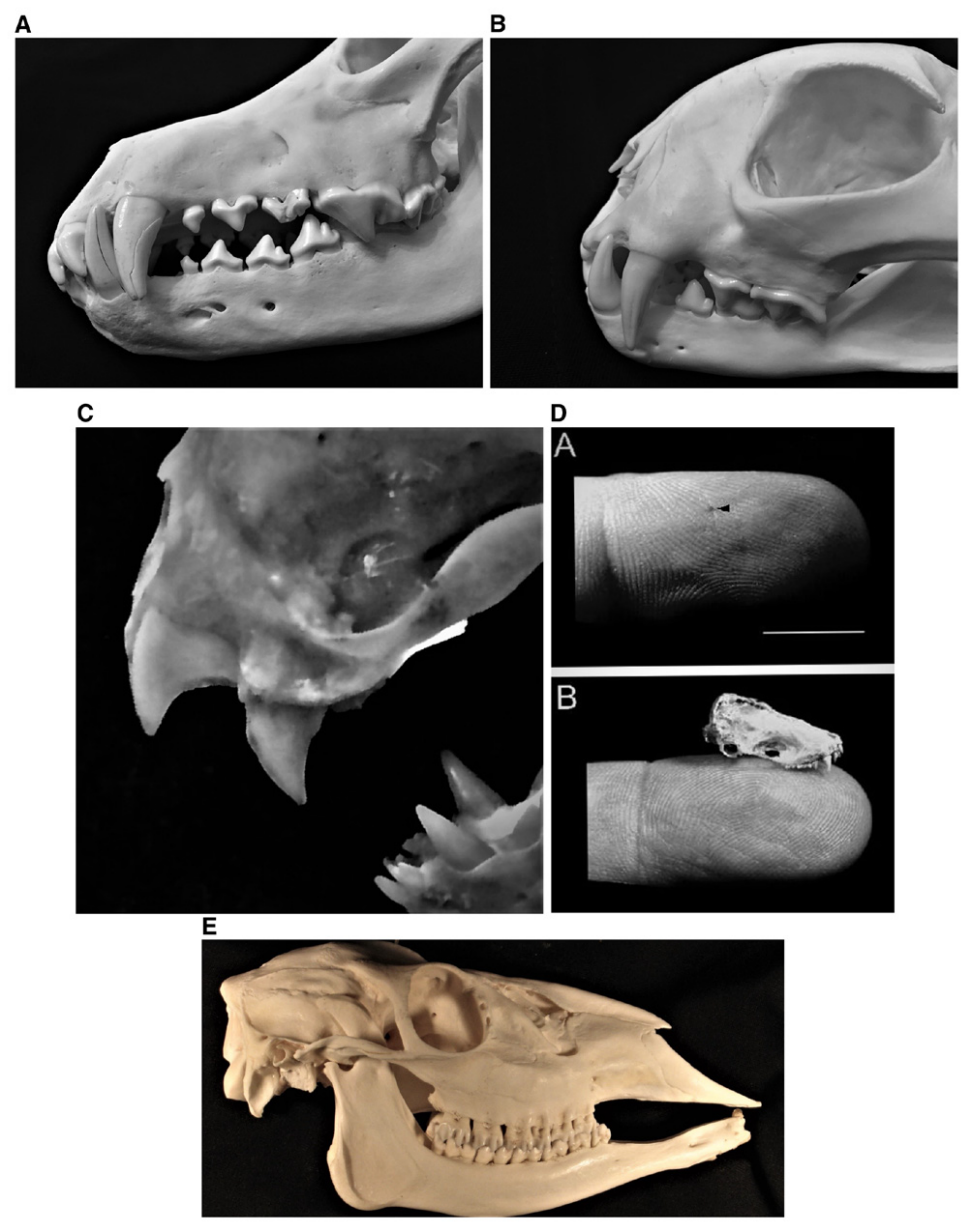
Lyssavirus virions are effectively transmitted transdermally via the saliva into peripheral tissues of a prospective host by the bites of infected mammals, as exemplified by representative mesocarnivores and bats (and in contrast to herbivores). **1A**. Close-up of the canines and carnassial teeth of a canid apex predator, the North American gray wolf, *Canis lupus.* **1B**. Close up of the canines and carnassial teeth of the most common rabid wild felid diagnosed within North America, the bobcat, *Lynx rufus.* **1C**. Close up of the specialized incisors and canines of the common vampire bat, *Desmodus rotundus.* **1D**. Example of an insectivorous bat bite. A. Demonstration of a typical small lesion to a finger from an insectivorous bat (bar inset approximately 1 cm). B. Comparison of the skull of an insectivorous bat to a human digit. This figure was reprinted with permission from Elsevier (Jackson AC, Fenton MB. Human rabies and bat bites. The Lancet. 2001. 357:1714)^201^. **1E**. Lateral view of the skull of a representative mammalian herbivore, demonstrating the distinct operational differences in the dentition (i.e. incisors, canines, and cheek teeth) between those taxa serving largely as “dead-end rabies victims” in contrast to typical lyssavirus reservoirs and vectors, depicted in **1A**–**D** (This figure was adapted from Smalette, specimen 12092010, Wikimedia Commons, the free media repository, licensed under the terms of Creative Commons Attribution-Share Alike 3.0 Unported license https://commons.wikimedia.org/wiki/File:12092010_Right_View.JPG#filelinks).

Early to end-stage clinical manifestations of encephalitis are generally recognized as key supportive factors in viral transmission, along the lines of mania^88^. However, even “normal”, daily social behaviors, involving mucosal or transdermal exposures, may also be operative towards routine perpetuation, as viral excretion in the saliva occurs days before the onset of abnormal signs. Morphologically, mammalian heterodont teeth have several different shapes and multiple functions, to bite, rip, grind, crush, groom, nip, shear, stab, suckle, etc. Beyond primary use in prey capture, killing, and feeding or for established specialized behaviors, the effectiveness of mammalian transmission of lyssaviruses via a bite may be better appreciated by cursorial examination of such teeth from a representative canid (Figure 1A), felid (Figure 1B), vampire bat (Figure 1C), and insectivorous bat (Figure 1D), designed for many different functions but secondarily repurposed as highly effective “pathogen delivery devices” into peripheral tissues. This feature is enhanced by inter-related characteristics, such as local muscular strength, bite force, physical dexterity, chewing capacities, etc., as basic life history attributes of most predators, in stark contrast to the feeding apparatus of a typical mammalian herbivore (Figure 1E). Such ultimate outcomes for highly successful intraspecific viral perpetuation appear obvious (e.g. rabid fox to fox, raccoon to raccoon, bat to bat, etc.), as well as for spillover opportunities to different species (e.g. rabid fox to deer, raccoon to woodchuck, bat to cow, etc.), with relevant public health, agricultural, and environmental ramifications^89–91^. For example, some of the highest case fatalities have occurred after human exposure to rabid wolves (likely infected originally by interactions with rabid dogs), given the risk for severe cranio-facial bites^92^. Also, while felids do not serve as typical reservoirs, they are highly efficient predators/vectors, presumably infected primarily during aggressive encounters with a bat, or via another mesocarnivore, such as a rabid dog, fox, raccoon, skunk, mongoose, ferret badger, etc., before a human or other species encounter^93^. In contrast to carnivores, vampire bats, as obligate vertebrate parasites, are the only mammals involved in natural viral exposures to other taxa directly because of their hematophagous nature, due to preying upon much larger livestock, humans, or other mammals^94^. For nearly all other, non-vampire bat–human encounters, individuals may not receive prophylaxis because of ignorance of the risk or because they may not realize they were exposed^95^. Although human infections with bat lyssaviruses have been documented on all continents, outside the Americas and Europe, this risk may not be appreciated more widely in Africa and Asia, where few human cases receive laboratory confirmation or characterization and the current epidemiological introspection is well focused upon the task of canine rabies remediation^1,2,4,77^.

Fundamentally, as nearly all rabies cases occur after a bite, applied epidemiological data collection on the incidence of animal bite by age, sex, season, locality, species, etc., can be highly informative to public health policy creation in mitigating risks of disease occurrence by preventing and responding to exposures^2,41,58,74,86,91,92,95,191^. Applied appropriately with mass dog vaccination and human prophylaxis in a local, national, or regional One Health context saves lives and healthcare costs^2,96^. For example, one multi-variate regression study in seven Latin American countries from 1995–2005 found that an increase in dog vaccinations decreased canine rabies cases, reported human exposures, and human deaths^8^. Within the Middle East, research incorporating proven epidemiological methods with phylogeographic approaches has shown the impact of environmental factors upon canine rabies virus dispersal^97^. Such anthropogenic facets have undoubtedly played a critical part since canine domestication to the present, given historical interdependence, translocations, and close animal–human bonds^1,2,84^. This increasing integration of classical epidemiological methodology with modern diagnostics, molecular techniques, health economics, or modeling approaches has provided key insights into applied rabies dynamics, improved prophylaxis, cost-effective prevention, and multi-species control strategies^2,59,98–110^ (Table 3).

**Table 3.** Selective illustration of a diversity of recent epidemiological applications in humans, domestic animals, and wildlife for improved detection, prevention, and control on a global basis.

Example	Citations
Informatics for policy making on human prophylaxis recommendations at a global level	98
Predictive modeling of potential spatial spread in a canine rabies-free continent	99
Annual animal rabies laboratory-based surveillance summary for North America	74
Emergency department syndrome-based surveillance	100
Meta-analysis of animal bite statistics in Iran	92
Using ecological insights to overcome barriers for improved canine vaccination	101
Geographic information system use for wildlife rabies outbreak response	102
Health economics comparison of canine rabies control demonstration sites in Africa and Asia	103
Public health investigation of mass human exposure events from bats in the USA	104
Cohort assessment of the risk of rabies in biting Haitian dogs	105
Retrospective, multi-hospital analysis of the relative adequacy of rabies immunoglobulin administration to patients	106
Prospective, spatiotemporal study of human exposure risk factors in Ethiopia	107
Cross-sectional household survey on dog populations, bite incidence, and rabies knowledge in an African community at risk	108
Case series of rare human rabies survivors in India	109
Human case report, after substantial patient contact with bats in the home, but without prophylaxis, demonstrating the need for continued education	110

## Modern strategies to prevent, control, and selectively eliminate rabies

Before the 20^th^ century, most global rabies prevention and control efforts focused upon a gamut of responses, including denial, avoiding exposures, quackery, quarantine, isolation, dog killing, collaring or muzzling, and wildlife culling, with varying levels of success. Thereafter, although some of these earlier strategies are still employed, a century of development has now resulted in pure, potent, safe, and effective rabies vaccines for administration to humans, domestic animals, and wildlife^2,111–113^. Somewhat unique for viral diseases, these vaccines may be used routinely for either pre-exposure prophylaxis (PrEP) or post-exposure prophylaxis (PEP) to minimize the opportunity for a productive lyssavirus infection (Table 4). Updated recommendations for human prophylaxis have been forthcoming, built upon epidemiological insights and clinical studies of biologics first licensed during the latter part of the 20^th^ century, focused upon greater dose-sparing use of the intradermal route of vaccine administration, shorter regimens, infiltration of rabies immunoglobulins (RIGs) or monoclonal antibodies, and relevant applications of PrEP to those at risk^2,10–12,14,19,98,114^. Greater harmonization of these guidelines is expected to follow suit in both LDCs and developed countries^115^. Unfortunately, despite highly effective PrEP and PEP, humans will still succumb to rabies because they receive no prophylaxis, a lack of RIGs or infiltration, inadequate vaccination, or a delay in PEP of several days or more, especially after a severe exposure. Historical and recent work highlights the importance of humoral immune responses and the role of VNAs directed against the viral G protein^113^. Although the development of new biologics is ongoing and the scrutiny to obtain vaccines directed against disparate lyssaviruses continues, the only approved products on the market are directed against rabies virus. Several new vaccine development concepts have been studied in animal models, including novel adjuvants, virus-like particles, and nucleic acid-, chimeric rabies virus-, simian adenovirus-, and epitope-based vaccines^50,192,193^. These approaches could expand the spectrum of coverage and may require even fewer vaccine doses or less-expensive applications for either human PrEP or PEP. Regardless of future innovations, rabies virus is the predominant lyssavirus of importance. As such, human survival is virtually assured by the prompt and proper use of today’s biologics after rabies virus exposure.

**Table 4.** Use of prophylaxis before or after lyssavirus exposure in humans, domestic animals, and wildlife^2,11,112^.

Group	Pre-exposure prophylaxis	Post-exposure prophylaxis
Humans	Parenteral vaccine doses administered to any persons at risk of viral exposure, with serological surveillance of certain occupational groups (i.e. laboratory workers, veterinarians, etc.) for determination of a routine booster when immunity wanes, based upon virus neutralization antibody detection	Thorough wound cleansing, infiltration of rabies immunoglobulin into wounds, and parenteral administration of several doses of rabies vaccine (for the previously vaccinated person, only rabies vaccine is administered)
Domestic animals	Ideally, all domestic animals (but especially dogs and cats) at risk of exposure should receive a single parenteral vaccine at around 3 months of age, a booster at about 1 year of age, and periodic annual or triennial boosters dependent upon label indications and local regulations	Immediate, single, parenteral re-vaccination upon known exposure to invoke an anamnestic response
Wildlife	Mesocarnivore reservoirs (e.g. coyotes, ferret badgers, foxes, jackals, mongoose, raccoons, raccoon dogs, etc.) may be targeted for oral vaccination by well-designed programs for which vaccine safety and efficacy have been determined (in addition, parenteral vaccination may occur for mammals maintained in zoological collections or by trap-vaccinate-release of free-ranging wild mammals)	Primarily occurs naturally when a previously vaccinated animal develops an anamnestic response upon consumption of another dose of oral vaccine

Equally impressive to the progress in the use of human prophylaxis is the success also demonstrated for animals^116–124^ (Table 5). All developed countries eliminated canine rabies. Increasingly, LDCs repeated the model, starting with the regional program in the Latin American countries, despite large numbers of free-ranging dogs in urban centers and rural communities. While the GEHRD can be accomplished via the combination of human prophylaxis and domestic animal control by vaccination, without the elimination of canine rabies virus circulation, the long-term effectiveness of such a strategy equates to the “incurable wound”. Rather, by removing the underlying problem, a primary rationale for most of the more-costly human prophylaxis is minimized. Such a vision is feasible, as the accumulated data from disease modeling studies indicate that the basic reproduction number, R_0_, is less than 2, control through mass canine vaccination is highly effective in reducing cases in dogs and subsequently in humans owing to a reduced animal burden, and ~70% annual coverage is a sound target for prevention^125,126^. Concerns about the estimated population of dogs to vaccinate, actual determination of vaccine coverage, relative density, age and birth rates, levels needed to prevent re-establishment from endemic areas, and more efficient methods of reaching free-ranging animals are identified as some of the crucial factors influencing the effectiveness of such interventions^125–128,194^.

**Table 5.** Evidence of global progress in applied rabies prevention, control, and elimination.

Locality	Interval	Item	Reference
India	2012–2016	Gradual estimated declines in human rabies cases within seven states (primarily because of human prophylaxis), with a need for improved surveillance at a national level	116
China	2007–2017	Decrease in estimated human cases from 3,300 to 516 (primarily because of human prophylaxis) at a national level, based on passive surveillance	117
Republic of Korea	1998 to date	Classified as a notifiable disease since 1961, with a decrease of 68 animal rabies cases to 0 by 2014, primarily by domestic animal vaccination and oral vaccination of wildlife	118
Thailand	1980–2015	Human rabies cases decreased from ~370 to ~5, concomitant with decline in animal cases	119
Vietnam	1992–2017	Reduced human-reported deaths from 404 to 74	120
Sri Lanka	1973–2015	With a national plan for elimination, human deaths declined from 377 to 24, while dog vaccinations increased from <300,000 to >1.4 million	121
KwaZulu-Natal, South Africa	2007–2014	Using a combination of methods, including increased public education, human prophylaxis, and dog vaccination, canine cases fell from 473 to 37 and human cases were reduced from approximately 9 to 0	122
Americas (21 Latin American and Caribbean countries)	1998–2014	Consistent decline in human and canine rabies case incidence, approaching 0	123
Europe	1978–2016	Based upon the European rabies surveillance data base, only 3,982 total animal cases were reported (an approximately 4.3-fold decrease) and at least 12 countries self-declared rabies freedom, primarily because of oral vaccination of wildlife	124

**Figure 2.**
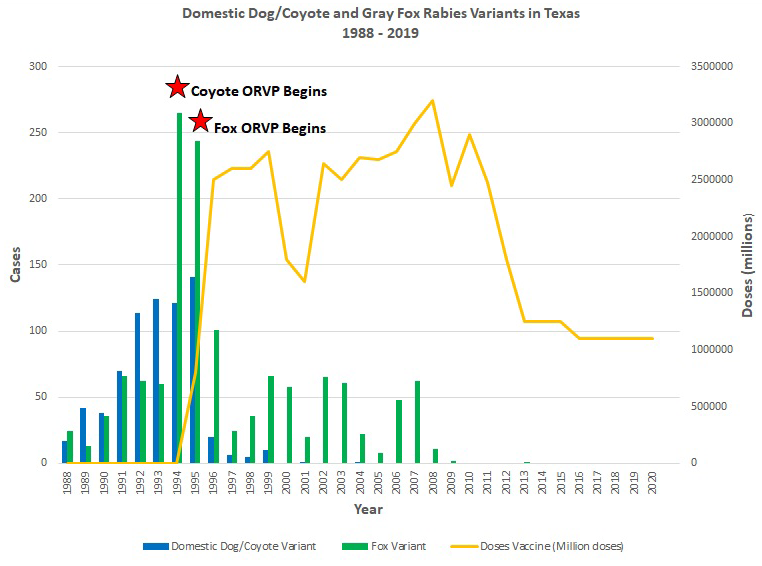
Demonstration of the impact of the Texas oral rabies vaccination program (ORVP) in response to coyote and gray fox rabies outbreaks. Comparison of laboratory-diagnosed cases of coyote and gray fox rabies virus variants to the combined number of oral rabies vaccine doses distributed over time in west-central and southern Texas, leading to elimination.

Beyond prevention in humans and domestic animals, rabies is the only zoonosis in which wildlife vaccination, using attenuated or recombinant biologics, has risen from an academic concept to a safe, effective, and economical long-term practice on a grand scale^129–133^. For example, after the multi-year use of oral rabies vaccine (ORV) distributed in edible baits, western Europe and large parts of southern Ontario became free of fox rabies^124,134^. Within the Republic of Korea, ORV was used for the elimination of rabies virus in raccoon dogs^132^. In the USA, ORV for raccoons began during 1990 and programs to date have prevented raccoon rabies spread beyond the eastern states as plans are formulated for elimination^135^. Locally, within the state of Texas, large outbreaks in coyotes and gray foxes occurred during the late 1980s and research began to evaluate the utility of ORV for these wild canids^136^. By the mid-1990s, large-scale ORV began in west-central Texas for gray foxes and southern Texas for coyotes (Figure 2). To date, tens of millions of baits have been distributed in the state over tens of millions of square kilometers. The last case of coyote rabies virus variant was detected during 2004 and the last case of gray fox rabies virus variant was diagnosed during 2013 (in an infected cow)^74^. Gray fox vaccination has ceased, with the elimination of that rabies virus variant, but, as a precaution, annual ORV maintenance occurs in southern Texas because of the threat of the re-emergence of coyote rabies. In concept, ORV programs could be expanded, based upon enhanced surveillance, particularly within Mexico and border locations^137^. Around the globe, additional species and biologics are under evaluation for ORV application^138–144^. Moreover, given the success of ORV in rabies suppression, other diseases are also being explored for prevention and control in wildlife^145–148^. Operational studies in different taxa will continue to uncover basic pathobiological and immunological mechanisms to improve upon the next generation of ORV^149^. Such advances stand as a legacy to those researchers from the 1960s who grappled with pragmatic ideas of how to approach the problem of wildlife reservoirs once canine rabies was prevented, controlled, or eliminated.

## Experimental treatment options

In comparison to rabies vaccines and antibodies for humans, anti-viral strategies have also been under development, with renewed fervor post-2004 and documentation of the first survivor without a history of vaccination, yet with much less discernable clinical progress, in part because of the obstacle of safe and effective delivery of compounds to the central nervous system^55,150,151^. Fundamentally, unlike the former focus on biologics, designed to prevent a productive infection, the latter efforts wrestle primarily with the “post-infection treatment” dilemma when routine public health interventions fail, and a clinical case develops. Recent investigations demonstrate that nucleoside inhibitors and their analogs, previously identified to inhibit other RNA viruses, are also capable of limiting lyssavirus replication. These could present potential broader-spectrum anti-viral candidates for future concentration. For example, a recent study demonstrated that favipiravir (T-705), a viral RNA-dependent RNA polymerase inhibitor that acts as a purine analog (shown previously to protect against filovirus infection), can limit lyssavirus infection *in vitro*^16^. Not surprisingly, observations of several such drug effects are not easily extrapolatable to *in vivo* data^152,153^. The likelihood of finding a lyssavirus-specific modality is predictably low, compared to the potential finesse of broader approaches for other “high-stakes” RNA virus targets among the Mononegavirales^154^. In the interim, this highly controversial aspect of the field will see-saw between an empirical, research-based *in vitro*/*in vivo* side and an emergency “hit-or-miss best guess” slant to clinical treatment of lyssavirus encephalitis, in which, among others, patient families, hospital administrators, multiple medical specialties, national regulators, ethicists, viral diagnosticians, and developers of novel biologics, as well as fit-for-purpose anti-viral drugs, will all play a part^2,42,55,150–155^. In addition, one part of the dilemma is the absence of relevant animal models to consider for actual clinical rabies treatment, particularly in a manner similar to the management of human encephalitis. However, there is no shortage of domestic animals that are euthanized after rabies virus exposure or the onset of compatible illness. Rather than euthanasia as the only current tool, perhaps these naturally occurring animal cases might be better utilized under more ideal circumstances. For example, as veterinarians receive PrEP, institutional intensive care facilities are available to isolate and sedate suspects safely, and academic teaching hospitals are renown for their biomedical research, greater progress in the field overall might ensue if the profession embarked upon the more routine clinical care and experimental treatment of rabid domestic animal patients, based upon current insights.

## Remaining issues

Given the palpable enthusiasm and actual progress generated by the GEHRD concept to date, candor and objectivity in the context of current pandemic events help to color expectations with the approach and passage of a ZBT world. What might this entail? As reflected in daily headlines, interconnected competing priorities will remain a reality, in contrast to “just rabies”. These include recognizable emergent communicable viral diseases (e.g. COVID-19 and zoonoses due to henipaviruses, hemorrhagic filoviruses, etc.), longstanding agricultural concerns over high-value commodities (e.g. ASF, FMDV, H1N1, etc.), devastating natural calamities, particularly associated with climate change (e.g. drought, fire, floods, etc.), and strong differing expert opinions about the “best way” to spend limited global funds in LDCs^156^. Even under ideal circumstances, a sustainable ZBT business plan remains elusive given wandering variables (i.e. the number of dogs at risk, the quantity of vaccine doses needed, the determination of producers to meet rising demands, the opportunity costs of local vs. regional sources of biologics, long-term sources of support, etc.), as do other unresolved topics^1,157,194,195^. Nevertheless, these are just anticipated nuances of a plan well in motion, and, as finer-grained program plans for GEHRD evolve, countries will grapple with these and other broader debates, such as a dependence upon external sources of modern biologics vs. a very clear need for self-sustainability (https://www.dawn.com/news/1526311/pakistan-to-become-self-sufficient-in-four-antisera-by-june).

One way to envision the landscape post-ZBT may be ascertained in part by scenarios whence this goal has already been achieved locally, nationally, or regionally^158^. Within this forum, some may toy with the somewhat unimaginable concept of “eradication” (i.e. quite dependent upon flexible terminology but in stark contrast to the obviousness patterned by the definition as pertinent to smallpox and rinderpest). Concomitant with such freedom is the flaunting by others of long-standing rules related to vaccination and the improper movement of adopted animals from LDCs that, not surprisingly, introduce rabies to previously canine rabies-free territories^159,160^. Similarly, a primary focus upon the threat of canine rabies translocation is warranted from enzootic to “free” areas, but ignoring the reality of wildlife rabies altogether seems an object legend for appreciation and disaster, using the experience of Taiwan alone^161^. Canine rabies deserves to be at the forefront, while wildlife rabies remains lurking prominently in the background. Understandably, while reservoirs such as foxes, mongooses, raccoons, and skunks have been recognized historically for many decades to centuries, such is not the case for ferret badgers, non-human primates, or other potential candidate hosts, especially in localities where lyssavirus surveillance is much less than ideal^162^.

As may be obvious from a cursorial examination of the more specialized aspects of the inarguable progress in rabies diagnostics and biologics, there is a certain skewedness towards technical approaches in disease problem-solving, with much less of a focus on more anthropological, economic, political, or societal concerns for introspection^163,195^. Without the inclusion of these arenas beyond mere “lip service”, transdisciplinary boundaries persist, preventing an ultimate understanding of why a given program may fail^164^. Additionally, “vaccine hesitancy” has crept even into the rabies field, both human and veterinary, despite an abundance of need, safety data, and epidemiological modeling (https://www.avma.org/javma-news/2020-03-01/vaccine-hesitancy). Unplanned exclusionary practices by discipline can undermine otherwise sophisticated solutions to long-term disease control, prevention, and elimination, unless there is a concerted effort to appreciate bias, conflict, distrust, xenophobia, polarization, and related administrative, community, cultural, and religious concerns^165^. In some situations, suppression of actionable healthcare priorities may not only be ignored but intentionally suppressed^166^. Besides public and political disparities, there are similar disconnects on continuing education needs and maintaining expertise among healthcare workers, veterinary staff, and often-overlooked wildlife professionals^167,168^. Also, the expressed routine needs of the applied user and the academic provider approach to trendy, fundable high-tech solutions does appear in need of remedy (as opposed to the simple, practical, available, and inexpensive, e.g. cell-phone technology, locally produced coolers to maintain the cold-chain, etc.)^169,188,196^.

One expectation of collateral damage from an unrelenting supply of naïve, unsupported, and alternative “facts” about nearly everything during the new internet age arises in part from the coverage and communications about rabies survivors and the misuse of simple terms, such as “treatment”. Clearly, rabies is a vaccine-preventable disease but is not treatable, per se^54^. One suggested downside may be a public misunderstanding of the ability to receive an outdated misnomer of “post-exposure treatment” after the onset of illness and thus being somewhat cavalier in reception to an accepted biomedical notion of prompt and proper PEP. While it is one thing having a true shortage in supply of biologics, living far from healthcare, or being poor in the pocket in the affordability of what should be otherwise provided for free as a life-saving intervention, it is yet quite another to be the receiver of exaggerated, misinterpreted, or false news about rabies. Similarly, a perceived long “event horizon” towards the recognition of an actual documented therapy coupled with the ongoing tragedy of human rabies that will continue for the foreseeable future underscore serious discussions over patient rights and individual options for euthanasia and a dignified death in the face of an obviously fatal outcome, unless more attractive alternatives beckon (https://timesofindia.indiatimes.com/india/can-rabies-patients-opt-for-euthanasia/articleshow/73164110.cms?utm_source=contentofinterest&utm_medium=text&utm_campaign=cppst).

## Conclusions and future directions

Lyssaviruses seem to attract special concentration compared to other members in the Rhabdoviridae, not the least of which is because these agents possess a zoonotic risk associated with the highest case fatality rate documented for any infectious disease. Besides stealth by modulation of replication locally and within the nervous system, lyssavirus proteins can effectively interact with host innate immunity and disable the establishment of otherwise resilient anti-viral responses. A fundamental understanding of this basic host–pathogen relationship at both the molecular and the cellular levels in multiple species and elucidating how non-traditional laboratory hosts, such as bats, might efficiently modulate lyssavirus infection under natural circumstances represent exciting challenges for future research. Such insights may open new avenues in the development of novel biologics and anti-viral strategies. These studies should lead to human and animal clinical trials, allowing the generation of new licensed vaccines, antibodies, drugs, and delivery systems that are even more efficient in the prevention or treatment of lyssavirus infection. Enhanced laboratory-based surveillance is key for human prophylaxis, domestic animal vaccination, wildlife management, program monitoring, and border controls. Otherwise, viral phenotypic plasticity combined with the broad distribution of known and suspected wild reservoirs, especially among mesocarnivores and bats, together with the likelihood of spillover to domestic animals, particularly dogs, raise the strong probability of enzootic perpetuation, epizootic spread, and translocation to “rabies-free” areas.

Over the past 10 years, substantial progress has occurred on a global level regarding pathogen discovery, diagnostics, prophylaxis, and engagement of professionals in academia, government, industry, and international non-governmental organizations. Further success requires maintaining this transdisciplinary philosophy, with collaboration among virologists, immunologists, epidemiologists, veterinarians, physicians, producers, regulators, economists, and social scientists within an updated One Health approach in a common endeavor to better understand, communicate, detect, prevent, control, and eliminate lyssavirus infections in the next decade^170–183^ (Table 6). Supporting focus, enthusiasm, and momentum, based upon the evidence at hand, is critical but will not be simple, overly rapid, or inexpensive (https://www.who.int/neglected_diseases/news/WHO-EB-commend-progress-against-NTDs-and-calls-roadmap-2021-2030/en/). These timely critical lessons learned about surveillance, diagnosis, and management, with best practices applied from one pathogen more than millennia-old in the making, should also be applicable to many of tomorrow’s emerging zoonoses. In this regard, the ensuing pandemic of SARS-CoV-2 presents a much-told cautionary tale as to a legacy related not only to rabies but also to other neglected tropical diseases as well (Table 7)^199,200^.

**Table 6.** Predictive scenarios for the rabies field over the next decade.

Likely events	Supportive citations
Broadened surveillance for new lyssavirus species among bats and other mammals	170
Prediction and documentation of associated mammalian species reservoir status for unresolved lyssaviruses (e.g. Mokola, Shimoni, etc.)	171
Better appreciation of bat reservoirs in suggestive “rabies-free” areas, such as islands	172
Refinement of linear flow and related assays for improved “point of care” use in the rapid diagnosis of lyssaviruses	173
Movement beyond pilot projects towards actual national canine rabies elimination in Asia	120
Demonstration of regional elimination of human rabies mediated via dogs in Africa	174
Clinical trials of new biologics to reduce or replace the use of rabies immunoglobulins	175
Expansion of human monoclonal antibodies breadth against divergent lyssaviruses	176
Licensing of purified, serum-free rabies vaccines, with updated label claims for intradermal use	177
Considerations on the use of oral rabies vaccines for other species, such as bats	178
Use of a single vaccine dose for pre-exposure vaccination in remote communities at risk	179
Abandonment of animal testing in the determination of vaccine potency	180,198
Evidence in support of anti-viral drug use based upon insight to viral targets	30
Programmatic use of oral vaccination of dogs for control among free-ranging animals	181
Elimination of canine rabies in Latin America and better “south-south” engagements for repetition of best practices beyond technology	182
“*In situ* genomic surveillance” expansion within lesser developed countries	183
Protection of “free regions” by expansive elimination of canine rabies in enzootic areas	184
Utilization of Gavi support for human rabies vaccination into national health programs	185

**Table 7.** Potential impacts of the COVID-19 pandemic upon rabies activities.

Benefits	Limitations
Greater appreciation for diseases of nature and viral zoonoses specifically, such as rabies	Lessons lost, due to pandemic fatigue
Enhanced laboratory-based surveillance for lyssaviruses	Pathogen discovery focused primarily upon coronaviruses alone
Additional scrutiny to better understand how bat populations deal with lyssavirus burden	Unnecessary backlash against bat populations in general
Broader consideration of dogs now as pets, rather than livestock for consumption, closure of wildlife markets, and halting use of bats as bushmeat	Unpopular consumptive activities driven ever more underground
Shelter-in-place, limiting human exposure to rabid animals	Mass unemployment drives even greater community shifts and increases individual movements for resources
More animals vaccinated in aftermath of best practices, including use of drive-up clinics	Veterinary services not viewed as an essential activity compared to public health
New vaccine approaches provide insights for novel human prophylaxis	Unfulfilled promises and adverse events sour demand for novel products
Anti-viral strategies reap extension against other RNA viruses, such as in the Mononegavirales	No major cross-reactivity to rhabdoviruses
Broader One Health adoptive strategies	Anti-public health sentiments due to presumption of civil liberties lost
Global elimination of human rabies mediated via dogs (GEHRD) achieved before 2030 owing to greater preventive focus	GEHRD setback for decades owing to economic global repercussions

## References

[1] Rupprecht C Kuzmin I Meslin F: Lyssaviruses and rabies: current conundrums, concerns, contradictions and controversies [version 1; peer review: 2 approved]. *F1000Res.* 2017; 6: 184. 10.12688/f1000research.10416.1 28299201 PMC5325067

[2] World Health Organization: WHO Expert Consultation on Rabies. Third Report. WHO TRS #1012, Geneva, Switzerland, 2019; 183 Reference Source

[3] Fooks AR: Conclusions Rabies. *Rev Sci Tech.* 2018; 37(2): 761–9. 10.20506/rst.37.2.2839 30747110

[4] Shipley R Wright E Selden D: Bats and Viruses: Emergence of Novel Lyssaviruses and Association of Bats with Viral Zoonoses in the EU. *Trop Med Infect Dis.* 2019; 4(1): 31. 10.3390/tropicalmed4010031 30736432 PMC6473451

[5] Kotait I Oliveira RdN Carrieri ML: Non-human primates as a reservoir for rabies virus in Brazil. *Zoonoses Public Health.* 2019; 66(1): 47–59. 10.1111/zph.12527 30288933

[6] Zhao JH Zhao LF Liu F: Ferret badger rabies in Zhejiang, Jiangxi and Taiwan, China. *Arch Virol.* 2019; 164(2): 579–84. 10.1007/s00705-018-4082-5 30417198

[7] World Health Organization: Laboratory techniques in rabies. 5th ed. WHO. Geneva, Switzerland. 2019 Reference Source

[8] Yoder J Younce E Lankester F: Healthcare demand in response to rabies elimination campaigns in Latin America. *PLoS Negl Trop Dis.* 2019; 13(9): e0007630. 10.1371/journal.pntd.0007630 31557160 PMC6762069

[9] Abela-Ridder B Balogh de K Kessels JA: Global rabies control: The role of international organisations and the Global Strategic Plan to eliminate dog-mediated human rabies. *Rev Sci Tech.* 2018; 37(2): 741–9. 10.20506/rst.37.2.2837 30747112

[10] Bharti OK Thakur B Rao R: Wound-only injection of rabies immunoglobulin (RIG) saves lives and costs less than a dollar per patient by "pooling strategy". *Vaccine.* 2019; 37 Suppl 1: A128–A131. 10.1016/j.vaccine.2019.07.087 31395454

[11] Sparrow E Torvaldsen S Newall AT: Recent advances in the development of monoclonal antibodies for rabies post exposure prophylaxis: A review of the current status of the clinical development pipeline. *Vaccine.* 2019; 37 Suppl 1: A132–A139. 10.1016/j.vaccine.2019.07.087 30503659

[12] Warrell MJ: Simplification of Rabies Postexposure Prophylaxis: A New 2-Visit Intradermal Vaccine Regimen. *Am J Trop Med Hyg.* 2019; 101(6): 1199–201. 10.4269/ajtmh.19-0252 31392953 PMC6896873

[13] Botto Nuñez G Becker DJ Plowright RK: The emergence of vampire bat rabies in Uruguay within a historical context. *Epidemiol Infect.* 2019; 147: e180. 10.1017/S0950268819000682 31063102 PMC6518465

[14] Kessels JA Recuenco S Navarro-Vela AM: Pre-exposure rabies prophylaxis: A systematic review. *Bull World Health Organ.* 2017; 95(3): 210–219C. 10.2471/BLT.16.173039 28250534 PMC5328107

[15] Poston R Hill R Allen C: Achieving scientific and regulatory success in implementing non-animal approaches to human and veterinary rabies vaccine testing: A NICEATM and IABS workshop report. *Biologicals.* 2019; 60: 8–14. 10.1016/j.biologicals.2019.06.005 31255474

[16] Yamada K Noguchi K Kimitsuki K: Reevaluation of the efficacy of favipiravir against rabies virus using *in vivo* imaging analysis. *Antiviral Res.* 2019; 172: 104641. 10.1016/j.antiviral.2019.104641 31672666

[17] Cliquet F Guiot AL Aubert M: Oral vaccination of dogs: A well-studied and undervalued tool for achieving human and dog rabies elimination. *Vet Res.* 2018; 49(1): 61. 10.1186/s13567-018-0554-6 30005701 PMC6045873

[18] Pan American Health Organization: Mexico is free from human rabies transmitted by dogs. Media Center, 2019 Reference Source

[19] Hampson K Ventura F Steenson R: The potential effect of improved provision of rabies post-exposure prophylaxis in Gavi-eligible countries: A modelling study. *Lancet Infect Dis.* 2019; 19(1): 102–11. 10.1016/S1473-3099(18)30512-7 30472178 PMC6300480

[20] Bertoune MAR Nickl B Krieger T: The phenotype of the RABV glycoprotein determines cellular and global virus load in the brain and is decisive for the pace of the disease. *Virology.* 2017; 511: 82–94. 10.1016/j.virol.2017.08.019 28841446

[21] Hossain MA Larrous F Rawlinson SM: Structural Elucidation of Viral Antagonism of Innate Immunity at the STAT1 Interface. *Cell Rep.* 2019; 29(7): 1934–1945.e8. 10.1016/j.celrep.2019.10.020 31722208

[22] Zaeck L Potratz M Freuling CM: High-Resolution 3D Imaging of Rabies Virus Infection in Solvent-Cleared Brain Tissue. *J Vis Exp.* 2019; (146). 10.3791/59402 31107452

[23] Ogino M Gupta N Green TJ: A dual-functional priming-capping loop of rhabdoviral RNA polymerases directs terminal *de novo* initiation and capping intermediate formation. *Nucleic Acids Res.* 2019; 47(1): 299–309. 10.1093/nar/gky1058 30395342 PMC6326812

[24] Riedel C Vasishtan D Pražák V: Cryo EM structure of the rabies virus ribonucleoprotein complex. *Sci Rep.* 2019; 9(1): 9639. 10.1038/s41598-019-46126-7 31270364 PMC6610074

[25] Zhang Y Wang Y Feng Y: Proteomic Profiling of Purified Rabies Virus Particles. *Virol Sin.* 2020; 35(2): 143–55. 10.1007/s12250-019-00157-6 31429011 PMC7090697

[26] Jespersen NE Leyrat C Gérard FC: The LC8-RavP ensemble Structure Evinces A Role for LC8 in Regulating Lyssavirus Polymerase Functionality. *J Mol Biol.* 2019; 431: 4959–77. 10.1016/j.jmb.2019.10.011 31634467 PMC7060403

[27] Luo J Zhang Y Zhang Q: The Deoptimization of Rabies Virus Matrix Protein Impacts Viral Transcription and Replication. *Viruses.* 2019; 12(1): 4. 10.3390/v12010004 31861477 PMC7019236

[28] Belot L Albertini A Gaudin Y: Structural and cellular biology of rhabdovirus entry. *Adv Virus Res.* 2019; 104: 147–83. 10.1016/bs.aivir.2019.05.003 31439148

[29] Nikolic J Lagaudrière-Gesbert C Scrima N: Structure and Function of Negri Bodies. *Adv Exp Med Biol.* 2019; 1215: 111–27. 10.1007/978-3-030-14741-9_6 31317498

[30] Horwitz JA Jenni S Harrison SC: Structure of a rabies virus polymerase complex from electron cryo-microscopy. *Proc Natl Acad Sci U S A.* 2020; 117(4): 2099–107. 10.1073/pnas.1918809117 31953264 PMC6995008

[31] Hellert J Buchrieser J Larrous F: Structure of the prefusion-locking broadly neutralizing antibody RVC20 bound to the rabies virus glycoprotein. *Nat Commun.* 2020; 11(1): 596. 10.1038/s41467-020-14398-7 32001700 PMC6992781

[32] Yang F Lin S Ye F: Structural Analysis of Rabies Virus Glycoprotein Reveals pH-Dependent Conformational Changes and Interactions with a Neutralizing Antibody. *Cell Host Microbe.* 2020; 27(3): 441–453.e7. 10.1016/j.chom.2019.12.012 32004500

[33] Davis BM Rall GF Schnell MJ: Everything You Always Wanted to Know About Rabies Virus (But Were Afraid to Ask). *Annu Rev Virol.* 2015; 2(1): 451–71. 10.1146/annurev-virology-100114-055157 26958924 PMC6842493

[34] Potratz M Zaeck L Christen M: Astrocyte Infection during Rabies Encephalitis Depends on the Virus Strain and Infection Route as Demonstrated by Novel Quantitative 3D Analysis of Cell Tropism. *Cells.* 2020; 9(2): 412. 10.3390/cells9020412 32053954 PMC7072253

[35] Dietzschold B Morimoto K Hooper DC: Genotypic and phenotypic diversity of rabies virus variants involved in human rabies: Implications for postexposure prophylaxis. *J Hum Virol.* 2000; 3(1): 50–7. 10774807

[36] Kammouni W Wood H Jackson AC: Lyssavirus phosphoproteins increase mitochondrial complex I activity and levels of reactive oxygen species. *J Neurovirol.* 2017; 23(5): 756–62. 10.1007/s13365-017-0550-z 28681345

[37] Sundaramoorthy V Green D Locke K: Novel role of SARM1 mediated axonal degeneration in the pathogenesis of rabies. *PLoS Pathog.* 2020; 16(2): e1008343. 10.1371/journal.ppat.1008343 32069324 PMC7048299

[38] Khalsi F Ayari A Romdhane MB: Rabies encephalitis in children: A resurgence of a fatal anthropozoonosis. *Afr Health Sci.* 2018; 18(3): 539–41. 10.4314/ahs.v18i3.10 30602985 PMC6307015

[39] Zhao H Zhang J Cheng C: Rabies Acquired through Mucosal Exposure, China, 2013. *Emerging Infect Dis.* 2019; 25(5): 1028–9. 10.3201/eid2505.181413 31002064 PMC6478212

[40] Lu XX Zhu WY Wu GZ: Rabies virus transmission via solid organs or tissue allotransplantation. *Infect Dis Poverty.* 2018; 7(1): 82. 10.1186/s40249-018-0467-7 30107857 PMC6092857

[41] Ghosh S Rana MS Islam MK: Trends and clinico-epidemiological features of human rabies cases in Bangladesh 2006–2018. *Sci Rep.* 2020; 10(1): 2410. 10.1038/s41598-020-59109-w 32051481 PMC7016137

[42] Jackson AC: Rabies: A medical perspective. *Rev Sci Tech.* 2018; 37(2): 569–80. 10.20506/rst.37.2.2825 30747124

[43] Farahtaj F Alizadeh L Gholami A: Natural Infection with Rabies Virus: A Histopathological and Immunohistochemical Study of Human Brains. *Osong Public Health Res Perspect.* 2019; 10(1): 6–11. 10.24171/j.phrp.2019.10.1.03 30847265 PMC6396821

[44] Bokade CM Gajimwar VS Meshram RM: Survival of Atypical Rabies Encephalitis. *Ann Indian Acad Neurol.* 2019; 22(3): 319–21. 10.4103/aian.AIAN_202_18 31359946 PMC6613421

[45] Jackson AC Del Bigio MR: Reader Response: Rabies encephalitis presenting with new-onset refractory status epilepticus (NORSE). *Neurol Clin Pract.* 2018; 8(5): 370–371. 10.1212/CPJ.0000000000000542 30564485 PMC6276334

[46] Luo Z Li Y Zhou M: Toll-Like Receptor 7 Enhances Rabies Virus-Induced Humoral Immunity by Facilitating the Formation of Germinal Centers. *Front Immunol.* 2019; 10: 429. 10.3389/fimmu.2019.00429 30906301 PMC6418000

[47] Katz ISS Guedes F Fernandes ER: Immunological aspects of rabies: A literature review. *Arch Virol.* 2017; 162(11): 3251–68. 10.1007/s00705-017-3484-0 28726129

[48] Li C Zhang H Ji L: Deficient Incorporation of Rabies Virus Glycoprotein into Virions Enhances Virus-Induced Immune Evasion and Viral Pathogenicity. *Viruses.* 2019; 11(3): 218. 10.3390/v11030218 30836694 PMC6466124

[49] Gold S Donnelly CA Nouvellet P: Rabies virus-neutralising antibodies in healthy, unvaccinated individuals: What do they mean for rabies epidemiology? *PLoS Negl Trop Dis.* 2020; 14(2): e0007933. 10.1371/journal.pntd.0007933 32053628 PMC7017994

[50] Ertl HCJ: Human immune responses to traditional and novel rabies vaccines. * Rev Sci Tech.* 2018; 37(2): 649–56. 10.20506/rst.37.2.2830 30747120

[51] Franka R Wallace R: Rabies diagnosis and surveillance in animals in the era of rabies elimination. * Rev Sci Tech.* 2018; 37(2): 359–70. 10.20506/rst.37.2.2807 30747142

[52] Dacheux L Bourhy H: Diagnostic tests for human rabies. *Rev Sci Tech.* 2018; 37(2): 581–93. 10.20506/rst.37.2.2826 30747123

[53] Gourlaouen M Angot A Mancin M: An inter-laboratory trial as a tool to increase rabies diagnostic capabilities of Sub-Saharan African Veterinary laboratories. *PLoS Negl Trop Dis.* 2020; 14(2): e0008010. 10.1371/journal.pntd.0008010 32040472 PMC7010240

[54] Wilson, PJ Rohde RE Oertli EH: Rabies: Clinical Considerations and Exposure Evaluations. Elsevier Press, St. Louis, Missouri, USA. 2019 Reference Source

[55] Smith SP Wu G Fooks AR: Trying to treat the untreatable: Experimental approaches to clear rabies virus infection from the CNS. *J Gen Virol.* 2019; 100(8): 1171–86. 10.1099/jgv.0.001269 31237530

[56] Gigante CM Dettinger L Powell JW: Multi-site evaluation of the LN34 pan-lyssavirus real-time RT-PCR assay for post-mortem rabies diagnostics. *PLoS One.* 2018; 13(5): e0197074. 10.1371/journal.pone.0197074 29768505 PMC5955534

[57] Davis AJ Nelson KM Kirby JD: Rabies Surveillance Identifies Potential Risk Corridors and Enables Management Evaluation. *Viruses.* 2019; 11(11): 1006. 10.3390/v11111006 31683632 PMC6893774

[58] Undurraga EA Meltzer MI Tran CH: Cost-Effectiveness Evaluation of a Novel Integrated Bite Case Management Program for the Control of Human Rabies, Haiti, 2014–2015. *Am J Trop Med Hyg.* 2017; 96(6): 1307–17. 10.4269/ajtmh.16-0785 28719253 PMC5462564

[59] Brunker K Nadin-Davis S Biek R: Genomic sequencing, evolution and molecular epidemiology of rabies virus. *Rev Sci Tech.* 2018; 37(2): 401–8. 10.20506/rst.37.2.2810 30747139

[60] de Thoisy B Bourhy H Delaval M: Bioecological Drivers of Rabies Virus Circulation in a Neotropical Bat Community. *PLoS Negl Trop Dis.* 2016; 10(1): e0004378. 10.1371/journal.pntd.0004378 26808820 PMC4726525

[61] de Rocha SM Oliveira SV Heinemann MB: Epidemiological Profile of Wild Rabies in Brazil (2002–2012). *Transbound Emerg Dis.* 2017; 64(2): 624–33. 10.1111/tbed.12428 26423323

[62] Huettmann F Magnuson EE Hueffer K: Ecological niche modeling of rabies in the changing Arctic of Alaska. *Acta Vet Scand.* 2017; 59(1): 18. 10.1186/s13028-017-0285-0 28320440 PMC5359834

[63] Nadin-Davis SA Fehlner-Gardiner C Gilbert AT: Origins of the arctic fox variant rabies viruses responsible for recent cases of the disease in southern Ontario. *PLoS Negl Trop Dis.* 2019; 13(9): e0007699. 10.1371/journal.pntd.0007699 31490919 PMC6750613

[64] Fisher CR Streicker DG Schnell MJ: The spread and evolution of rabies virus: Conquering new frontiers. *Nat Rev Microbiol.* 2018; 16(4): 241–55. 10.1038/nrmicro.2018.11 29479072 PMC6899062

[65] Hampson K Coudeville L Lembo T: Estimating the global burden of endemic canine rabies. *PLoS Negl Trop Dis.* 2015; 9(4): e0003709. 10.1371/journal.pntd.0003709 25881058 PMC4400070

[66] Rupprecht CE Bannazadeh Baghi H Del Rio Vilas VJ: Historical, current and expected future occurrence of rabies in enzootic regions. *Rev Sci Tech.* 2018; 37(2): 729–39. 10.20506/rst.37.2.2836 30747113

[67] Harrist A Styczynski A Wynn D: Human Rabies — Wyoming and Utah, 2015. *MMWR Morb Mortal Wkly Rep.* 2016; 65(21): 529–33. 10.15585/mmwr.mm6521a1 27253630

[68] Johnson N Montano Hirose JA: The impact of paralytic bovine rabies transmitted by vampire bats in Latin America and the Caribbean. *Rev Sci Tech.* 2018; 37(2): 451–9. 10.20506/rst.37.2.2814 30747135

[69] Khismatullina NA Gulyukin AM Gulyukin MI: [Two cases of hydrophobia in the Republic of Tatarstan: *In vivo* and postmortem laboratory diagnosis]. *Vopr Virusol.* 2015; 60(2): 18–24. 26182652

[70] Taxitiemuer A Tuerdi G Zhang Y: An Investigation of the First Case of Human Rabies Caused by a Fox in China in May 2016. *Biomed Environ Sci.* 2017; 30(11): 825–8. 10.3967/bes2017.110 29216959

[71] Yang DK Kim HH Lee KK: Mass vaccination has led to the elimination of rabies since 2014 in South Korea. *Clin Exp Vaccine Res.* 2017; 6(2): 111–9. 10.7774/cevr.2017.6.2.111 28775975 PMC5540959

[72] Huang J Ruan S Shu Y: Modeling the Transmission Dynamics of Rabies for Dog, Chinese Ferret Badger and Human Interactions in Zhejiang Province, China. *Bull Math Biol.* 2019; 81(4): 939–62. 10.1007/s11538-018-00537-1 30536160

[73] Birhane MG Cleaton JM Monroe BP: Rabies surveillance in the United States during 2015. *J Am Vet Med Assoc.* 2017; 250(10): 1117–30. 10.2460/javma.250.10.1117 28467751

[74] Ma X Monroe BP Cleaton JM: Public Veterinary Medicine: Public Health: Rabies surveillance in the United States during 2018. *J Am Vet Med Assoc.* 2020; 256(2): 195–208. 10.2460/javma.256.2.195 31910075

[75] Styczynski A Tran C Dirlikov E: Human Rabies - Puerto Rico, 2015. *MMWR Morb Mortal Wkly Rep.* 2017; 65(52): 1474–6. 10.15585/mmwr.mm6552a4 28056006

[76] Mani RS Sundara Raju YG Ramana PV: Human rabies following a non-human primate bite in India. *J Travel Med.* 2016; 23(3): taw007. 10.1093/jtm/taw007 26988199

[77] Markotter W Coertse J: Bat lyssaviruses. *Rev Sci Tech.* 2018; 37(2): 385–400. 10.20506/rst.37.2.2809 30747140

[78] Marston DA Banyard AC McElhinney LM: The lyssavirus host-specificity conundrum-rabies virus-the exception not the rule. *Curr Opin Virol.* 2018; 28: 68–73. 10.1016/j.coviro.2017.11.007 29182939

[79] Faria NR Suchard MA Rambaut A: Simultaneously reconstructing viral cross-species transmission history and identifying the underlying constraints. * Philos Trans R Soc Lond B Biol Sci.* 2013; 368(1614): 20120196. 10.1098/rstb.2012.0196 23382420 PMC3678322

[80] Nadin-Davis S Alnabelseya N Knowles MK: The phylogeography of *Myotis* bat-associated rabies viruses across Canada. *PLoS Negl Trop Dis.* 2017; 11(5): e0005541. 10.1371/journal.pntd.0005541 28542160 PMC5453604

[81] Escobar LE Peterson AT Favi M: Bat-borne rabies in Latin America. *Rev Inst Med Trop Sao Paulo.* 2015; 57(1): 63–72. 10.1590/S0036-46652015000100009 25651328 PMC4325525

[82] Kuzmina NA Lemey P Kuzmin IV: The phylogeography and spatiotemporal spread of south-central skunk rabies virus. *PLoS One.* 2013; 8(12): e82348. 10.1371/journal.pone.0082348 24312657 PMC3849458

[83] Ding NZ Xu DS Sun YY: A permanent host shift of rabies virus from *Chiroptera* to *Carnivora* associated with recombination. *Sci Rep.* 2017; 7(1): 289. 10.1038/s41598-017-00395-2 28325933 PMC5428239

[84] Troupin C Dacheux L Tanguy M: Large-Scale Phylogenomic Analysis Reveals the Complex Evolutionary History of Rabies Virus in Multiple Carnivore Hosts. *PLoS Pathog.* 2016; 12(12): e1006041. 10.1371/journal.ppat.1006041 27977811 PMC5158080

[85] Gilbert AT: Rabies virus vectors and reservoir species. *Rev Sci Tech.* 2018; 37(2): 371–84. 10.20506/rst.37.2.2808 30747141

[86] Hikufe EH Freuling CM Athingo R: Ecology and epidemiology of rabies in humans, domestic animals and wildlife in Namibia, 2011–2017. *PLoS Negl Trop Dis.* 2019; 13(4): e0007355. 10.1371/journal.pntd.0007355 30990805 PMC6486109

[87] Vora NM Orciari LA Niezgoda M: Clinical management and humoral immune responses to rabies post-exposure prophylaxis among three patients who received solid organs from a donor with rabies. *Transpl Infect Dis.* 2015; 17(3): 389–95. 10.1111/tid.12393 25851103 PMC4642444

[88] Brookes VJ Dürr S Ward MP: Rabies-induced behavioural changes are key to rabies persistence in dog populations: Investigation using a network-based model. *PLoS Negl Trop Dis.* 2019; 13(9): e0007739. 10.1371/journal.pntd.0007739 31545810 PMC6776358

[89] Theimer TC Dyer AC Keeley BW: Ecological Potential for Rabies Virus Transmission via Scavenging of Dead Bats by Mesocarnivores. *J Wildl Dis.* 2017; 53(2): 382–5. 10.7589/2016-09-203 28094609

[90] Lobo D DeBenedet C Fehlner-Gardiner C: Raccoon rabies outbreak in Hamilton, Ontario: A progress report. *Can Commun Dis Rep.* 2018; 44(5): 116–21. 10.14745/ccdr.v44i05a05 31007622 PMC6449115

[91] Pieracci EG Pearson CM Wallace RM: *Vital Signs*: Trends in Human Rabies Deaths and Exposures - United States, 1938-2018. *MMWR Morb Mortal Wkly Rep.* 2019; 68(23): 524–8. 10.15585/mmwr.mm6823e1 31194721 PMC6613553

[92] Abedi M Doosti-Irani A Jahanbakhsh F: Epidemiology of animal bite in Iran during a 20-year period (1993–2013): A meta-analysis. *Trop Med Health.* 2019; 47: 55. 10.1186/s41182-019-0182-5 31798312 PMC6884825

[93] Soler-Rangel S Jiménez-Restrepo N Nariño D: Rabies encephalitis and extra-neural manifestations in a patient bitten by a domestic cat. *Rev Inst Med Trop Sao Paulo.* 2020; 62: e1. 10.1590/S1678-9946202062001 31967209 PMC6968790

[94] Rocha F Dias RA: The common vampire bat *Desmodus rotundus* (Chiroptera: Phyllostomidae) and the transmission of the rabies virus to livestock: A contact network approach and recommendations for surveillance and control. *Prev Vet Med.* 2020; 174: 104809. 10.1016/j.prevetmed.2019.104809 31756671

[95] Dato VM Campagnolo ER Long J: Systematic Review of Human Bat Rabies Virus Variant Cases: Evaluating Unprotected Physical Contact with Claws and Teeth in Support of Accurate Risk Assessments. *PLoS One.* 2016; 11(7): e0159443. 10.1371/journal.pone.0159443 27459720 PMC4961291

[96] Purwo Suseno P Rysava K Brum E: Lessons for rabies control and elimination programmes: A decade of One Health experience from Bali, Indonesia. *Rev Sci Tech.* 2019; 38(1): 213–224. 10.20506/rst.38.1.2954 31564729 PMC7612388

[97] Dellicour S Troupin C Jahanbakhsh F: Using phylogeographic approaches to analyse the dispersal history, velocity and direction of viral lineages - Application to rabies virus spread in Iran. *Mol Ecol.* 2019; 28(18): 4335–50. 10.1111/mec.15222 31535448

[98] Hampson K Abela-Ridder B Bharti O: Modelling to inform prophylaxis regimens to prevent human rabies. *Vaccine.* 2019; 37(Suppl 1): A166–A173. 10.1016/j.vaccine.2018.11.010 30528846 PMC7612382

[99] Johnstone-Robertson SP Fleming PJS Ward MP: Predicted Spatial Spread of Canine Rabies in Australia. *PLoS Negl Trop Dis.* 2017; 11(1): e0005312. 10.1371/journal.pntd.0005312 28114327 PMC5289603

[100] Bemis K Frias M Patel MT: Using an Emergency Department Syndromic Surveillance System to Evaluate Reporting of Potential Rabies Exposures, Illinois, 2013–2015. *Public Health Rep.* 2017; 132(1_suppl): 59S–64S. 10.1177/0033354917708355 28692394 PMC5676512

[101] Schildecker S Millien M Blanton JD: Dog Ecology and Barriers to Canine Rabies Control in the Republic of Haiti, 2014–2015. *Transbound Emerg Dis.* 2017; 64(5): 1433–42. 10.1111/tbed.12531 27313170

[102] Giannakopoulos A Valiakos G Papaspyropoulos K: Rabies outbreak in Greece during 2012–2014: Use of Geographical Information System for analysis, risk assessment and control. *Epidemiol Infect.* 2016; 144(14): 3068–79. 10.1017/S0950268816001527 27435434 PMC9150405

[103] Elser JL Hatch BG Taylor LH: Towards canine rabies elimination: Economic comparisons of three project sites. *Transbound Emerg Dis.* 2018; 65: 135–45. 10.1111/tbed.12637 28299897

[104] Hsu CH Brown CM Murphy JM: Perceptions and Practices of Mass Bat Exposure Events in the Setting of Rabies Among U.S. Public Health Agencies. *Zoonoses Public Health.* 2017; 64(2): 127–36. 10.1111/zph.12289 27389926 PMC5525325

[105] Medley AM Millien MF Blanton JD: Retrospective Cohort Study to Assess the Risk of Rabies in Biting Dogs, 2013⁻2015, Republic of Haiti. *Trop Med Infect Dis.* 2017; 2(12): 14. 10.3390/tropicalmed2020014 30270873 PMC6082081

[106] Hwang GS Rizk E Bui LN: Adherence to guideline recommendations for human rabies immune globulin patient selection, dosing, timing, and anatomical site of administration in rabies postexposure prophylaxis. *Hum Vaccin Immunother.* 2020; 16(1): 51–60. 10.1080/21645515.2019.1632680 31210569 PMC7012082

[107] Gebru G Romha G Asefa A: Risk Factors and Spatio-Temporal Patterns of Human Rabies Exposure in Northwestern Tigray, Ethiopia. *Ann Glob Health.* 2019; 85(1): 119. 10.5334/aogh.2518 31517464 PMC6743033

[108] Mbilo C Kabongo JB Pyana PP: Dog Ecology, Bite Incidence, and Disease Awareness: A Cross-Sectional Survey among a Rabies-Affected Community in the Democratic Republic of the Congo. *Vaccines (Basel).* 2019; 7(3): 98. 10.3390/vaccines7030098 31454908 PMC6789516

[109] Mani RS Damodar T Divyashree S: Case Reports: Survival from Rabies: Case Series from India. *Am J Trop Med Hyg.* 2019; 100(1): 165–9. 10.4269/ajtmh.18-0711 30398147 PMC6335910

[110] Peterson D Barbeau B McCaffrey K: Human Rabies - Utah, 2018. *MMWR Morb Mortal Wkly Rep.* 2020; 69(5): 121–4. 10.15585/mmwr.mm6905a1 32027626 PMC7004398

[111] Manning SE Rupprecht CE Fishbein D: Human rabies prevention--United States, 2008: Recommendations of the Advisory Committee on Immunization Practices. *MMWR Recomm Rep.* 2008; 57(RR-3): 1–28. 18496505

[112] Brown CM Slavinski S Ettestad P: Compendium of Animal Rabies Prevention and Control, 2016. *J Am Vet Med Assoc.* 2016; 248(5): 505–17. 10.2460/javma.248.5.505 26885593

[113] Banyard AC McElhinney LM Johnson N: History of rabies control by vaccination. *Rev Sci Tech.* 2018; 37(2): 305–22. 10.20506/rst.37.2.2804 30747146

[114] Pattanaik A Mani RS: WHO's new rabies recommendations: Implications for high incidence countries. *Curr Opin Infect Dis.* 2019; 32(5): 401–406. 10.1097/QCO.0000000000000578 31305491

[115] O’ Leary ST Maldonado YA Kimberlin DW: Update from the Advisory Committee on Immunization Practices. *J Pediatric Infect Dis Soc.* 2019; 8(6): 495–500. 10.1093/jpids/piz058 31589289

[116] Sudarshan MK Ashwath Narayana DH: Appraisal of surveillance of human rabies and animal bites in seven states of India. *Indian J Public Health.* 2019; 63(Supplement): S3–S8. 10.4103/ijph.IJPH_377_19 31603084

[117] Qian MB Chen J Bergquist R: Neglected tropical diseases in the People’s Republic of China: Progress towards elimination. *Infect Dis Poverty.* 2019; 8(1): 86. 10.1186/s40249-019-0599-4 31578147 PMC6775666

[118] Yang DK Cho IS Kim HH: Strategies for controlling dog-mediated human rabies in Asia: Using ‘ One Health’ principles to assess control programmes for rabies. *Rev Sci Tech.* 2018; 37(2): 473–481. 10.20506/rst.37.2.2816 30747133

[119] World Health Organization: Towards a rabies-free Thailand by 2020. Media reporting. Geneva, Switzerland. 2017 Reference Source

[120] Nguyen HTT Nguyen HT Nguyen TTT: Progress towards rabies control and elimination in Vietnam. *Rev Sci Tech.* 2019; 38(1): 199–212. 10.20506/rst.38.1.2953 31564730

[121] Rahman SA Isloor S: Rabies on the Indian subcontinent. *Rev Sci Tech.* 2018; 37(2): 529–42. 10.20506/rst.37.2.2821 30747128

[122] LeRoux K Stewart D Perrett KD: Rabies control in KwaZulu-Natal, South Africa. *Bull World Health Organ.* 2018; 96(5): 360–5. 10.2471/BLT.17.194886 29875521 PMC5985419

[123] Freire de Carvalho M Vigilato MAN Pompei JA: Rabies in the Americas: 1998–2014. *PLoS Negl Trop Dis.* 2018; 12(3): e0006271. 10.1371/journal.pntd.0006271 29558465 PMC5877887

[124] Müller FT Freuling CM: Rabies control in Europe: An overview of past, current and future strategies. *Rev Sci Tech.* 2018; 37(2): 409–19. 10.20506/rst.37.2.2811 30747138

[125] Cleaveland S Thumbi SM Sambo M: Proof of concept of mass dog vaccination for the control and elimination of canine rabies. *Rev Sci Tech.* 2018; 37(2): 559–68. 10.20506/rst.37.2.2824 30747125 PMC7612386

[126] Rattanavipapong W Thavorncharoensap M Youngkong S: The impact of transmission dynamics of rabies control: Systematic review. *Vaccine.* 2019; 37 Suppl 1: A154–A165. 10.1016/j.vaccine.2018.11.035 30528329

[127] Leung T Davis SA: Rabies Vaccination Targets for Stray Dog Populations. *Front Vet Sci.* 2017; 4: 52. 10.3389/fvets.2017.00052 28451589 PMC5389970

[128] Jeon S Cleaton J Meltzer MI: Determining the post-elimination level of vaccination needed to prevent re-establishment of dog rabies. *PLoS Negl Trop Dis.* 2019; 13(12): e0007869. 10.1371/journal.pntd.0007869 31790398 PMC6907870

[129] Mähl P Cliquet F Guiot AL: Twenty year experience of the oral rabies vaccine SAG2 in wildlife: A global review. *Vet Res.* 2014; 45(1): 1. 10.1186/s13567-014-0077-8 25106552 PMC4423639

[130] Cliquet F Picard-Meyer E Mojzis M: In-Depth Characterization of Live Vaccines Used in Europe for Oral Rabies Vaccination of Wildlife. *PLoS One.* 2015; 10(10): e0141537. 10.1371/journal.pone.0141537 26509266 PMC4625003

[131] Maki J Guiot AL Aubert M: Oral vaccination of wildlife using a vaccinia-rabies-glycoprotein recombinant virus vaccine (RABORAL V-RG ®): a global review. *Vet Res.* 2017; 48(1): 662. 10.1186/s13567-017-0459-9 28938920 PMC5610451

[132] Yang DK Kim HH Cho IS: Strategies to maintain Korea's animal rabies non-occurrence status. *Clin Exp Vaccine Res.* 2018; 7(2): 87–92. 10.7774/cevr.2018.7.2.87 30112347 PMC6082677

[133] Head JR Vos A Blanton J: Environmental distribution of certain modified live-virus vaccines with a high safety profile presents a low-risk, high-reward to control zoonotic diseases. *Sci Rep.* 2019; 9(1): 6783. 10.1038/s41598-019-42714-9 31043646 PMC6494895

[134] Fehlner-Gardiner C: Rabies control in North America - past, present and future. *Rev Sci Tech.* 2018; 37(2): 421–37. 10.20506/rst.37.2.2812 30747137

[135] Elmore SA Chipman RB Slate D: Management and modeling approaches for controlling raccoon rabies: The road to elimination. *PLoS Negl Trop Dis.* 2017; 11(3): e0005249. 10.1371/journal.pntd.0005249 28301480 PMC5354248

[136] Sidwa TJ Wilson PJ Moore GM: Evaluation of oral rabies vaccination programs for control of rabies epizootics in coyotes and gray foxes: 1995–2003. *J Am Vet Med Assoc.* 2005; 227(5): 785–92. 10.2460/javma.2005.227.785 16178403

[137] Garcés-Ayala F Aréchiga-Ceballos N Ortiz-Alcántara JM: Molecular characterization of atypical antigenic variants of canine rabies virus reveals its reintroduction by wildlife vectors in southeastern Mexico. *Arch Virol.* 2017; 162(12): 3629–37. 10.1007/s00705-017-3529-4 28819692

[138] Hsu AP Tseng CH Barrat J: Safety, efficacy and immunogenicity evaluation of the SAG2 oral rabies vaccine in Formosan ferret badgers. *PLoS One.* 2017; 12(10): e0184831. 10.1371/journal.pone.0184831 28977009 PMC5627901

[139] Zhugunissov K Bulatov Y Taranov D: Protective immune response of oral rabies vaccine in stray dogs, corsacs and steppe wolves after a single immunization. *Arch Virol.* 2017; 162(11): 3363–70. 10.1007/s00705-017-3499-6 28766059

[140] Hassel R Vos A Clausen P: Experimental screening studies on rabies virus transmission and oral rabies vaccination of the Greater Kudu ( *Tragelaphus strepsiceros*). *Sci Rep.* 2018; 8(1): 16599. 10.1038/s41598-018-34985-5 30413745 PMC6226427

[141] Ortmann S Kretzschmar A Kaiser C: *In Vivo* Safety Studies With SPBN GASGAS in the Frame of Oral Vaccination of Foxes and Raccoon Dogs Against Rabies. *Front Vet Sci.* 2018; 5: 91. 10.3389/fvets.2018.00091 29868616 PMC5968751

[142] Wohlers A Lankau EW Oertli EH: Challenges to controlling rabies in skunk populations using oral rabies vaccination: A review. *Zoonoses Public Health.* 2018; 65(4): 373–85. 10.1111/zph.12471 29633545

[143] Koeppel KN Kuhn BF Thompson PN: Oral bait preferences for rabies vaccination in free-ranging black-backed jackal (*Canis mesomelas*) and non-target species in a multi-site field study in a peri-urban protected area in South Africa. *Prev Vet Med.* 2020; 175: 104867. 10.1016/j.prevetmed.2019.104867 31927421

[144] Berentsen AR Ellis CK Johnson SR: Immunogenicity of Ontario Rabies Vaccine for Small Indian Mongooses (*Herpestes auropunctatus*). *J Wildl Dis.* 2020; 56(1): 224–228. 31567036

[145] Rossi S Staubach C Blome S: Controlling of CSFV in European wild boar using oral vaccination: A review. *Front Microbiol.* 2015; 6: 1141. 10.3389/fmicb.2015.01141 26557109 PMC4615961

[146] Kern A Zhou CW Jia F: Live-vaccinia virus encapsulation in pH-sensitive polymer increases safety of a reservoir-targeted Lyme disease vaccine by targeting gastrointestinal release. *Vaccine.* 2016; 34(38): 4507–13. 10.1016/j.vaccine.2016.07.059 27502570 PMC4996687

[147] Lesellier S Boschiroli ML Barrat J: Detection of live M. bovis BCG in tissues and IFN-γ responses in European badgers (*Meles meles*) vaccinated by oropharyngeal instillation or directly in the ileum. *BMC Vet Res.* 2019; 15(1): 445. 10.1186/s12917-019-2166-4 31810466 PMC6898942

[148] Flies AS Flies EJ Fox S: An oral bait vaccination approach for the Tasmanian devil facial tumor diseases. *Expert Rev Vaccines.* 2020; 19(1): 1–10. 10.1080/14760584.2020.1711058 31971036

[149] Te Kamp V Freuling CM Vos A: Responsiveness of various reservoir species to oral rabies vaccination correlates with differences in vaccine uptake of mucosa associated lymphoid tissues. *Sci Rep.* 2020; 10(1): 2919. 10.1038/s41598-020-59719-4 32076025 PMC7031338

[150] Koraka P Martina B: Antivirals for human use against rabies and prospects for their future application. *Rev Sci Tech.* 2018; 37(2): 673–80. 10.20506/rst.37.2.2832 30747118

[151] Du Pont V Plemper RK Schnell MJ: Status of antiviral therapeutics against rabies virus and related emerging lyssaviruses. *Curr Opin Virol.* 2019; 35: 1–13. 10.1016/j.coviro.2018.12.009 30753961 PMC6556400

[152] Anindita PD Sasaki M Okada K: Ribavirin-related compounds exert *in vitro* inhibitory effects toward rabies virus. *Antiviral Res.* 2018; 154: 1–9. 10.1016/j.antiviral.2018.03.011 29601893

[153] Rogée S Larrous F Jochmans D: Pyrimethamine inhibits rabies virus replication *in vitro*. *Antiviral Res.* 2019; 161: 1–9. 10.1016/j.antiviral.2018.10.016 30367894

[154] Latorre V Mattenberger F Geller R: Chaperoning the *Mononegavirales*: Current Knowledge and Future Directions. *Viruses.* 2018; 10(12): 699. 10.3390/v10120699 30544818 PMC6315898

[155] Willoughby RE Jr: Rabies: Rare Human Infection - Common Questions. *Infect Dis Clin North Am.* 2015; 29(4): 637–50. 10.1016/j.idc.2015.07.006 26384549

[156] Malarski M Hasso-Agopsowicz M Soble A: Vaccine impact on antimicrobial resistance to inform Gavi, the Vaccine Alliance’s 2018 Vaccine Investment Strategy: report from an expert survey [version 1; peer review: 2 approved]. *F1000Res.* 2019; 8: 1685. 10.12688/f1000research.20100.1 31737260 PMC6807152

[157] Wallace RM Undurraga EA Blanton JD: Elimination of Dog-Mediated Human Rabies Deaths by 2030: Needs Assessment and Alternatives for Progress Based on Dog Vaccination. *Front Vet Sci.* 2017; 4: 9. 10.3389/fvets.2017.00009 28239608 PMC5300989

[158] Robardet E Bosnjak D Englund L: Zero Endemic Cases of Wildlife Rabies (Classical Rabies Virus, RABV) in the European Union by 2020: An Achievable Goal. *Trop Med Infect Dis.* 2019; 4(4): 124. 10.3390/tropicalmed4040124 31575054 PMC6958318

[159] Hercules Y Bryant NJ Wallace RM: Rabies in a Dog Imported from Egypt - Connecticut, 2017. *MMWR Morb Mortal Wkly Rep.* 2018; 67(50): 1388–91. 10.15585/mmwr.mm6750a3 30571670 PMC6342549

[160] Cliquet F Wasniewski M: Maintenance of rabies-free areas. *Rev Sci Tech.* 2018; 37(2): 691–702. 10.20506/rst.37.2.2834 30747116

[161] Lan YC Wen TH Chang CC: Indigenous Wildlife Rabies in Taiwan: Ferret Badgers, a Long Term Terrestrial Reservoir. *Biomed Res Int.* 2017; 2017: 5491640. 10.1155/2017/5491640 28497055 PMC5405374

[162] Coertse J Markotter W Le Roux K: New isolations of the rabies-related Mokola virus from South Africa. *BMC Vet Res.* 2017; 13(1): 37. 10.1186/s12917-017-0948-0 28143485 PMC5282659

[163] Bardosh KL: Towards a science of global health delivery: A socio-anthropological framework to improve the effectiveness of neglected tropical disease interventions. *PLoS Negl Trop Dis.* 2018; 12(7): e0006537. 10.1371/journal.pntd.0006537 30024887 PMC6053127

[164] Attema AE He L Cook AJC: Unbiased assessment of disease surveillance utilities: A prospect theory application. *PLoS Negl Trop Dis.* 2019; 13(15): e0007364. 10.1371/journal.pntd.0007364 31042708 PMC6513105

[165] Aenishaenslin C Brunet P Lévesque F: Understanding the Connections Between Dogs, Health and Inuit Through a Mixed-Methods Study. *EcoHealth.* 2019; 16(1): 151–60. 10.1007/s10393-018-1386-6 30552532

[166] Briggs CL: Uncovering a tragic flaw in revolutionary health policies: From health and communicative inequities to communicative justice in health. *Salud Colect.* 2017; 13(3): 411–27. 10.18294/sc.2017.1152 29340509

[167] Tarrant S Grewal J Yaglom H: Zoonotic Disease Exposure Risk and Rabies Vaccination Among Wildlife Professionals. *EcoHealth.* 2020; 17(1): 74–83. 10.1007/s10393-020-01469-w 31993824 PMC7219209

[168] Manoharan A Chellaiyan VG Madhusudan M: Effect of educational intervention on the knowledge of rabies among medical school students of Chennai. *J Educ Health Promot.* 2019; 8: 208. 10.4103/jehp.jehp_161_19 31807598 PMC6852282

[169] Neevel AMG Hemrika T Claassen E: A research agenda to reinforce rabies control: A qualitative and quantitative prioritization. *PLoS Negl Trop Dis.* 2018; 12(5): e0006387. 10.1371/journal.pntd.0006387 29727444 PMC5955568

[170] Hsu WC Hsu CL Tu YC: Standard Operating Procedure for Lyssavirus Surveillance of the Bat Population in Taiwan. *J Vis Exp.* 2019; (150). 10.3791/59421 31524862

[171] Babayan SA Orton RJ Streicker DG: Predicting reservoir hosts and arthropod vectors from evolutionary signatures in RNA virus genomes. *Science.* 2018; 362(6414): 577–80. 10.1126/science.aap9072 30385576 PMC6536379

[172] Seetahal JFR Greenberg L Satheshkumar PS: The Serological Prevalence of Rabies Virus-Neutralizing Antibodies in the Bat Population on the Caribbean Island of Trinidad. *Viruses.* 2020; 12(2):178. 10.3390/v12020178 32033370 PMC7077287

[173] Klein A Fahrion A Finke S: Further Evidence of Inadequate Quality in Lateral Flow Devices Commercially Offered for the Diagnosis of Rabies. *Trop Med Infect Dis.* 2020; 5(1): 13. 10.3390/tropicalmed5010013 31963635 PMC7157750

[174] Athingo R Tenzin T Shilongo A: Fighting Dog-Mediated Rabies in Namibia— Implementation of a Rabies Elimination Program in the Northern Communal Areas. *Trop Med Infect Dis.* 2020; 5(1): 12. 10.3390/tropicalmed5010012 31963400 PMC7157552

[175] Plummer JR McGettigan JP: Incorporating B cell activating factor (BAFF) into the membrane of rabies virus (RABV) particles improves the speed and magnitude of vaccine-induced antibody responses. *PLoS Negl Trop Dis.* 2019; 13(11): e0007800. 10.1371/journal.pntd.0007800 31725816 PMC6855436

[176] de Benedictis P Minola A Rota Nodari E: Development of broad-spectrum human monoclonal antibodies for rabies post-exposure prophylaxis. *EMBO Mol Med.* 2016; 8(4): 407–21. 10.15252/emmm.201505986 26992832 PMC4818751

[177] Angsuwatcharakon P Khomvilai S Limsuwun K: Immunogenicity and safety of WHO-approved TRC-ID regimen with a chromatographically purified Vero cell rabies vaccine with or without rabies immunoglobulin in children. *Expert Rev Vaccines.* 2018; 17(2): 185–188. 10.1080/14760584.2018.1421074 29285961

[178] Bakker KM Rocke TE Osorio JE: Fluorescent biomarkers demonstrate prospects for spreadable vaccines to control disease transmission in wild bats. *Nat Ecol Evol.* 2019; 3(12): 1697–704. 10.1038/s41559-019-1032-x 31740844 PMC6887541

[179] Wang C Dulal P Zhou X: A simian-adenovirus-vectored rabies vaccine suitable for thermostabilisation and clinical development for low-cost single-dose pre-exposure prophylaxis. *PLoS Negl Trop Dis.* 2018; 12(10): e0006870. 10.1371/journal.pntd.0006870 30372438 PMC6224154

[180] Toinon A Moreno N Chausse H: Potency test to discriminate between differentially over-inactivated rabies vaccines: Agreement between the NIH assay and a G-protein based ELISA. *Biologicals.* 2019; 60: 49–54. 10.1016/j.biologicals.2019.05.004 31105021

[181] Gibson AD Mazeri S Yale G: Development of a Non-Meat-Based, Mass Producible and Effective Bait for Oral Vaccination of Dogs against Rabies in Goa State, India. *Trop Med Infect Dis.* 2019; 4(3): 118. 10.3390/tropicalmed4030118 31487795 PMC6789727

[182] Vigilato MAN Molina-Flores B Del Rio Vilas VJ: Canine rabies elimination: Governance principles. *Rev Sci Tech.* 2018; 37(2): 703–709. 10.20506/rst.37.2.2859 30747115

[183] Brunker K Jaswant G Thumbi SM: Rapid in-country sequencing of whole virus genomes to inform rabies elimination programmes [version 2; peer review: 3 approved]. *Wellcome Open Res.* 2020; 5: 3. 10.12688/wellcomeopenres.15518.2 32090172 PMC7001756

[184] Rupprecht CE Abela-Ridder B Abila R: Towards rabies elimination in the Asia-Pacific region: From theory to practice. *Biologicals.* 2020; 64: 83–95. 10.1016/j.biologicals.2020.01.008 32089431

[185] Sudarshan MK: Vision 2030: Dog-mediated human rabies-free India: Action must begin now. *Indian J Public Health.* 2017; 61(1): 1–2. 10.4103/ijph.IJPH_20_17 28218154

[186] Tian B Zhou M Yang Y: Lab-Attenuated Rabies Virus Causes Abortive Infection and Induces Cytokine Expression in Astrocytes by Activating Mitochondrial Antiviral-Signaling Protein Signaling Pathway. *Front Immunol.* 2018; 8: 2011. 10.3389/fimmu.2017.02011 29403485 PMC5785723

[187] Pattabhi S Knoll ML Gale M: DHX15 Is a Coreceptor for RLR Signaling That Promotes Antiviral Defense Against RNA Virus Infection. *J Interferon Cytokine Res.* 2019; 39(6): 331–46. 10.1089/jir.2018.0163 31090472 PMC6590726

[188] Cleaton JM Wallace RM Crowdis K: Impact of community-delivered SMS alerts on dog-owner participation during a mass rabies vaccination campaign, Haiti 2017. *Vaccine.* 2018; 36(17): 2321–2325. 10.1016/j.vaccine.2018.03.017 29580642 PMC6066789

[189] Salin P Blondel D Kerkerian-Le Goff L: Golgi staining-like retrograde labeling of brain circuits using rabies virus: Focus onto the striatonigral neurons. *J Neurosci Methods.* 2020; 344: 108872. 10.1016/j.jneumeth.2020.108872 32693000

[190] John B Kumar S Kumar S: Child survivor of rabies in India: a case report. *Paediatr Int Child Health.* 2020; 1–6. 10.1080/20469047.2020.1785198 32744918

[191] Whitehouse ER Peterson D McCaffrey K: Evaluation of Online Risk Assessment To Identify Rabies Exposures Among Health Care Workers - Utah, 2019. *MMWR Morb Mortal Wkly Rep.* 2020; 69(29): 956–959. 10.15585/mmwr.mm6929a3 32701943 PMC7377820

[192] Napolitano F Merone R Abbate A: A next generation vaccine against human rabies based on a single dose of a chimpanzee adenovirus vector serotype C. *PLoS Negl Trop Dis.* 2020; 14(7): e0008459. 10.1371/journal.pntd.0008459 32667913 PMC7363076

[193] Fisher CR Lowe DE Smith TG: Lyssavirus Vaccine with a Chimeric Glycoprotein Protects across Phylogroups. *Cell Rep.* 2020; 32(3): 107920. 10.1016/j.celrep.2020.107920 32697993 PMC7373069

[194] Mbilo C Coetzer A Bonfoh B: Dog rabies control in West and Central Africa: A review. *Acta Trop.* 2020; 105459. 10.1016/j.actatropica.2020.105459 32404295

[195] Castillo-Neyra R Buttenheim AM Brown J: Behavioral and structural barriers to accessing human post-exposure prophylaxis and other preventive practices in Arequipa, Peru, during a canine rabies epidemic. *PLoS Negl Trop Dis.* 2020; 14(7): e0008478. 10.1371/journal.pntd.0008478 32692739 PMC7394441

[196] Lugelo A Hampson K Bigambo M: Controlling Human Rabies: The Development of an Effective, Inexpensive and Locally Made Passive Cooling Device for Storing Thermotolerant Animal Rabies Vaccines. *Trop Med Infect Dis.* 2020; 5(3): E130. 10.3390/tropicalmed5030130 32796605 PMC7558109

[197] Botto Nuñez G Becker DJ Lawrence RL: Synergistic Effects of Grassland Fragmentation and Temperature on Bovine Rabies Emergence. *Ecohealth.* 2020; 17(2): 203–216. 10.1007/s10393-020-01486-9 32699950 PMC7885335

[198] Sanchez N Soulet D Bonnet E: Rabies Vaccine Characterization by Nanoparticle Tracking Analysis. *Sci Rep.* 2020; 10(1): 8149. 10.1038/s41598-020-64572-6 32424186 PMC7235079

[199] Nachega JB Mbala-Kingebeni P Otshudiema J: Responding to the Challenge of the Dual COVID-19 and Ebola Epidemics in the Democratic Republic of Congo-Priorities for Achieving Control. *Am J Trop Med Hyg.* 2020; 103(2): 597–602. 10.4269/ajtmh.20-0642 32563272 PMC7410434

[200] Raynor B Díaz EW Shinnick J: The impact of the COVID-19 pandemic on rabies reemergence in Latin America: the case of Arequipa, Peru. Preprint. *medRxiv.* 2020; 2020.08.06.20169581. 10.1101/2020.08.06.20169581 34019548 PMC8174740

[201] Jackson AC Fenton MB: Human rabies and bat bites. *Lancet.* 2001; 357(9269): 1714. 10.1016/S0140-6736(00)04852-2 11428374

